# Bengal Report of the Epidemic Cholera

**Published:** 1832-07-01

**Authors:** 


**REPORT ON THE EPIDEMIC CHOLERA MORBUS, AS IT VISITED THE TERRITORIES SUBJECT TO THE PRESIDENCY OF BENGAL, IN THE YEARS 1817, 1818, AND 1819. Drawn up by Order of the Government, under the Superintendence of the Medical Board. By *James Jameson*, Assist. Surgeon and Secretary to the Board. Octavo, pp. 325. Calcutta, 1820.**

THE epidemic cholera made its first appearance in the division of our Indian possessions subject to the presidency of Bengal. But the Bengal Board were not the first to report upon the malady; on the contrary, the Medical Board of Bombay published their Report in the year 1818, whilst the present was deferred until 1820. This circumstance is important, as it serves to explain some inaccuracies and errors into which ourselves and others have fallen. On the strength of one statement, for instance, the Bombay Board asserted that the epidemic broke out first in Zilla Jessore, and radiated from thence to all parts of India. The Editor of this Journal was the first in this country to receive the Bombay Report from the hands of Dr. Steuart, who had drawn it up. He published all the statements it contained as authentic, and, thus disseminated, they have subsequently been copied into every work upon the subject, and adopted as the basis of medical reasoning. We shall presently see, how erroneous the common opinion on the origin of the disease has been proved by careful and accurate investigations.

The method adopted by the Bengal Board to procure information respecting the epidemic was this. A circular, specifying the points on which replies were desirable, was forwarded to all the individuals on the list of the Medical Department. Queries embracing the main topics of interest and importance—atmospheric influence and conditions, contagion, endemic influence, the time and mode of appearance of the disease at any place, its rise, progress, and decline, its mortality, its pathological characters, its treatment, its sequelæ, and so on, were specifically addressed to two hundred and thirty-eight individuals. One hundred and twenty-four only sent replies at all, a circumstance calculated to elicit, in our opinion, equal regret, surprise, and dissatisfaction from all who have the good of their fellow-men or the interests of the profession itself at heart. Why men should superciliously hold their tongues on such an occasion, we cannot so much as conceive; but let that pass. Twenty-four answered merely to state their inability, in any way, to assist the Board, in the proposed enquiry. In this perhaps many were right. The remaining hundred communicated much valuable information, the whole of which has been embodied in, and indeed has formed the Report of the Board. We should state here, that this differs from the reports of the Bombay and Madras Boards in one great respect. In the *latter* the individual reports, or copious extracts from them, are given in detail, and are merely preceded by a summary of the evidence on the part of the Board. In this (the Bengal) we have no individual reports, but the statements of all have been consolidated and fused into a systematic and consistent treatise. Each plan presents its advantages, and on the whole we are well satisfied that both have been adopted. The method that has been pursued is delineated in the following short quotation.

"It was therefore determined to abandon this method; and in its stead, to adopt the form of a digest or compendium of all the facts within reach. In doing this, the mode of describing diseases, usually adhered to by practical writers, has been followed, with as little deviation as possible; that is, the symptoms have been treated of first; then the causes, proximate, remote, predisposing and exciting; then the mortality caused by the disease; then some peculiarities observed by it; and lastly the method of cure. To the whole have been prefixed an introduction describing the rise and progress of the Epidemic; and a sketch of the Weather in Bengal, and in the Upper Provinces, during several years antecedent to its appearance.—*Pref.* x.

Mr. Jameson quotes many passages from ancient writers to prove that all the symptoms of the disease have been enumerated in former times. In our analysis of the Madras Report, and in our observations on the epidemic in the last number of this Journal, we have touched so freely on this subject, that we need not revert to it. It must require a vast amount of credulity or incredulity to assert or to imagine now, that the epidemic presents any new, undescribed, or pathognomonic symptoms by which it may be known from other described visitations of cholera. The man who could make such an assertion must be very ignorant, very obstinate, or very impudent. Nay, not only has the disease been delineated in all its essential characters and conditions, but no doubt can exist of its having raged as an EPIDEMIC in India, if not elsewhere. Thus, in the Spring of 1781, an epidemic malady, characterized by dreadful spasms of the extremities and trunk, with distressing vomiting and purging, laid hold of a detachment five thousand strong, under Colonel Pearse, in the Northern Circars. Men dropped down by dozens, and even those less severely affected were generally dead or past recovery in less than an hour. Death raged in the camp, the road was strewed with the dying and the dead, and not less than seven hundred men were cut off. The mode of attack, the persons attacked, and the progress of the attack were precisely similar to what has been latterly observed during the present epidemic. But the disease did not cease after disabling Colonel Pearse's detachment. It passed southward, reached Calcutta, occasioned for a fortnight a very great mortality, then passed northward, and, owing to the imperfection of the documents preserved, its course can be traced no longer. The disease then has undoubtedly existed, nay, has been described as an epidemic. Then in what does the epidemic cholera before us and upon us differ from former recorded visitations? Simply in this, that its sphere has been more extended, its influence more deadly, its might more irresistible, than we have known them heretofore. We have no symptom, no cadaveric characters, no general nor partial laws, no phenomena about it or within it, which can enable us to say that it is a new disease, a malady which had never previously been witnessed, experienced, or described. To deny this is to deny undisputed facts and fly in the face of all philosophic reasoning —it is to make assertion superior to proof—to prefer dogmatic prejudice to authentic records. On the part of those who know no better, this is folly; in those who have the means of reference and consultation, it is worse, it is delusion, wilful delusion.

This introduction to the Report occupies, with the preface which we have been considering, 68 pages. The former is chiefly occupied with remarks on the condition of the seasons in Bengal, previous to the eruption of the epidemic. It appears that great and unusual atmospheric vicissitudes, or rather alterations, had commenced, and that they continued till after the epidemic had made some progress. For a description of the unnatural character of the seasons, we must refer such of our readers as are curious on the matter, to the Report; it is enough for our present purpose that such was the case. As might be expected, the human constitution, as well as the thermometer, felt the changes. Throughout 1816, low fevers predominated, and Indostan was visited by the infectious malignant sore throat, a disease previously known there only by name. In the upper Provinces, bilious remittent fever raged epidemically, and occasioned such mortality, as surpassed "any thing on record in the medical annals of Bengal." The military stations wore a gloom hardly to be imagined—the visits of families were almost put a stop to, and the few that were made were those of condolence for departed relatives and friends—in many Native villages, the whole population was ill, and shops were shut for want of people to attend them—the banks of the rivers were at all times covered with the dying and the dead—in Cutch and in Sindh, several cities were said to be so depopulated, that the living were unable to bury the dead, a circumstance that even De Foe denies to have occurred in the great plague in London—and throughout upper Indostan, the horned cattle were so sickly, that their bodies could be seen strewed in vast numbers in the pastures by passing travellers. Such was the state of public health in India in 1816. The cholera had not yet appeared. The previous seasons had been most unnatural, and we presume had been most unwholesome; at all events, man and cattle died, and Native and European were smitten with the epidemic maladies.

> Hic quondam morbo cœli miseranda coorta est
> Tempestas, totoque Autumni incanduit aestu,
> Et genus omne neci pecudum dedit, omne ferarum,
> Corrupitque lacus, infecit pabula tabo.

The cold season of 1816-17, brought, not health and pleasure, azure skies, elasticity and vigour to animal nature, freshness and verdure to the plant of the garden and grass of the plain. It brought not these its natural gifts, but mist and fog and sultry winds, the appurtenances of an Indian Autumn, not a Winter. Now, however, European and Native were healthy, there was a sort of pause, a breathing time between the pestilential fever that had just been, and the mightier pestilence that was about to be. February, March, April of 1817 presented "singular deviations" from their ordinary course—the rains set in twenty days earlier than usual, on the 25th of May—this month, with June, July, August, were extraordinarily wet, "an immense quantity" of rain falling, the river being quite full, the country generally under water, and the previous comparative healthiness giving way to fever, hepatitis, flux, and acute rheumatism. In August the Natives for the first time began to suffer severely from the epidemic cholera. The consideration of the seasons is pursued with patience and ability by Mr. Jameson as far as the month of May, 1819. We may state generally that the variations to which we have already referred, and for a more particular and precise knowledge of which we must direct those who wish for it to the original Report, continued until the latter month, when southerly winds and settled weather set in, and the cholera withdrew from Bengal. It is singular that on two occasions, in February 1818 and in April 1819, sudden changes of temperature appeared to revive the epidemic which had previously been dormant and declining.

> "This sketch of the weather being now brought to a conclusion, some apology may be necessary for its prolixity and minuteness of detail.—It might, indeed, have been wished, to make it less dry and tedious.—But it was only by entering largely into particulars, and by minutely following the successive variations of each period, and thus affording the reader an opportunity of comparing them with the fluctuations in the healthiness of those periods, that we could hope to trace a connexion between the generation of the prevailing pestilence, and the preceding irregularities in the season. How far this has been successfully done, must be left to the reader's judgment, when he shall have perused the account of the rise of the epidemic now about to be given.—This, at least, he will not doubt, that there was a remarkable coincidence between the extraordinary irregularities manifested throughout India in 1816 and 1817, and the rise of the epidemic; and that its subsequent abatements and revivals, were in some measure dependent on corresponding vicissitudes in the state of the weather." lxviii.

We have thought it necessary to devote some little attention to the atmospheric constitution of India, or at least of Bengal, prior to the out-break of the epidemic. It is undoubtedly true that, since its establishment, the latter has in some degree defied all seasons and conquered all climates. But it is no less consistent with experience and with common sense, that circumstances may exert great power in exciting a disease, which, when it is in being, exert but a feeble influence upon it. Thus cold applied to the surface of the body will excite pneumonia, but this being induced the removal of the cold is not followed by a removal of its original consequence. The difference between the cases is this, that in the former we have a general disposition to disease created, in the latter an insulated malady. Whether the irregularity of the seasons was the cause of the epidemic cholera, or whether it was not, it is certain and indisputable that irregularity did prevail to a very unusual extent, that epidemic fevers of a grave description occurred in the latter part of 1816, that the unnatural character of the seasons continued, and that in 1817 the epidemic made its appearance. These are facts, let men found what doctrines upon them fancy or reason may dictate. We proceed to the section on the rise and progress of the disease.

Cholera morbus has prevailed more or less endemically during the hot and rainy seasons of every successive year, in the lower Provinces of Hindostan. But previously to 1817, its influence was limited, and its sphere circumscribed. During the first six months of 1817 it shewed itself sooner, and was more common than in former years. In Nuddea and other districts, it would seem to have occurred in an unusual degree in May and June. On the 28th of August, it was reported to the Government that the disease had suddenly appeared epidemically in Jessore, a populous town situated in the centre of the delta of the Ganges, that it was committing extraordinary ravages, and that its inhabitants were flying in crowds from the place.

"So little was the nature of the new pestilence yet understood; and such was the extreme consternation produced by it, that the Civil Courts of the District were shut; and a stop put for a time to business of every description. Although the general emigration which took place from the city, would seem to have had a decidedly beneficial effect on the state of its health, by diminishing that density of population, which has been since invariably found to be a powerful auxiliary to the Epidemic; yet such was the energy of the disease in this its first onset, and so fatally destructive was it of human life, that in this district alone, it is reported to have, within the space of a few weeks, cut off more than six thousand of the inhabitants.

It is necessary to be thus particular regarding the breaking out of the disorder at Jessore; because from the alarming nature of the circumstances, which attended its appearance in that quarter; connected with its rapid and general spread as an Epidemic over almost every portion of the Lower Provinces, accompanying or immediately following that appearance; an idea then arose, and has since obtained very general belief, that Jessore was the place, in which the disease primarily originated, and whence, as from a focus, its pestilential virus, of whatever nature, emanated, to the surrounding districts. What served to give validity to this conjecture, was an opinion then entertained, and since industriously propagated, that the *fomes*, or specific poison producing the disease, had its rise, not in any vitiated state of the atmosphere, or other cause of general operation, but in circumstances of a purely local nature: such as the use of rancid fish and blighted grain.

It is nevertheless certain, that nothing could be more erroneous than this notion of the local origin of the Epidemic. For, not to speak of its frequent occurrence, so early as May in some parts of Nuddea, and other districts already adverted to, it is quite clear from the statements of the medical staff, written separately and without interchange of knowledge or communication, that, more than a month previously to Jessore's becoming affected, the disease had begun to prevail epidemically in the distant Provinces of Behar and Dacca; and that before the expiration of the first week in August, it had firmly established itself in many other parts of Bengal. Thus it is distinctly stated to have broken out in the City of Patna on the 11th of July; to have spread to the contiguous station of Dinapore, and the adjacent villages early in August; and to have remained without intermission in that neighbourhood, until the end of January following.* In like manner, after having in July appeared at Sunergong, a Town on the banks of a branch of the great river Megna, it thence proceeded, visiting the *ghauts* or public ferries and grain markets in its way, to Nuraingunge and Dacca, where it arrived in the beginning of August. During the whole of July and August, eight out of eighteen Police Departments into which zila Kishnagur or Nuddeea, on the east side of the Hoogly, is divided, were also fully subject to its influence; and it had about the middle of the latter month even found its way to the remote Province of Sylhet, which is separated from the eastern parts of

---

* "It appears from accounts received since this sketch was drawn up, that the disease had ravaged Nuseerabad, a town in Momensing, in June; and had even largely affected the South Eastern Division of that district, in the early part of the month or last days of May: following the course of the Burumpooter; and irregularly attacking the villages on its banks."

Bengal Proper by the great Rivers Ganges and Burumpooter. On the 23d of August we find it raging at Chittagong, far round the Eastern corner of the Bay of Bengal; at the same moment in Rajshahy, a centrical district lying East of the Ganges; and not a week afterwards, in the high and distant tracts of Bhaugulpore and Monghyr. The exact date of its appearance in Calcutta has not been ascertained.—But there is little doubt, that it visited some spots of the town and suburbs as early as the beginning of August; that it daily gained ground, and before the end of the month had widely spread its ravages, in a manner threatening to sweep off a large portion of the native population; and that in the early part of September, even the European portion of the community was no longer secure from the concentrated activity of the poison.

These facts are more than sufficient to shew the fallacy of every theory, which attempts to derive the disease from any local source; or to trace it to any one particular spot, as the centre from which it was emitted to the surrounding countries.—They prove, without the possibility of dispute, that it broke out at very remote places at one and the same time, or at the distance of such short intervals, as to establish the impossibility of the pestilential virus being, in this stage of its progress, propagated by contagion, or any of the other known modes of successive production; and that its general diffusion was therefore referable to some cause of more universal operation.*

Soon after the middle of September, the disease, now strictly epidemical, extended itself in every direction; within the short space of a few weeks stretching from the most easterly parts of Poorneea, Dinagepore, and Sylhet, to the extreme borders of Balasore and Cuttack; and reaching from the mouths of the Ganges nearly as high as its junction with the Jumna.

Within the area of several thousand miles, thus in so short a period brought under its influence, few towns or villages of any considerable size wholly escaped its attacks; almost every spot being, notwithstanding the great irregularity of its course, and waywardness of its approach, sooner or later, and in a greater or less degree, subjected to its dreadful visitations.—The cities of Dacca and Patna, the towns of Balasore, Burrisaul, Burdwan, Rungpore, Malda, Bhaugulpore, Chupra, and Moozufferpore, with the Military stations of Monghyr, Buxar, and Ghazeepore, all suffered severely: and throughout the whole extent of the Delta of the Ganges, and more especially in the tracts bordering on the Hoogly and the Jellinghy Rivers, so great was the mortality, that the bulk of the whole population was sensibly diminished by the dreadful ravages of the distemper. It is remarkable, that the large and populous city of Moorshedabad, from extent and local position apparently very favourably circumstanced for the attacks of the Epidemic, should have escaped with comparatively little loss, whilst all around was so severely scourged." 9.

---

* "A few dates may be marked of the principal places attacked in the early part of the progress of the Epidemic, according to the order of their succession. —May and June. One *Thanna* or police Division of Kishnagur; and Mymensing generally. July. Eight Divisions of the former district; Sunergong in the Dacca District; and on the 11th Patna. August. In the first week Calcutta, Dacca and Dinapore; about the middle, Nattore; on the 17th, Sylhet; on the 19th, Jessore; and towards the end of the month, Bhaugulpore and Monghyr. September 15th. Balasore, Burisaul, Burdwan; 17th, Buxar; 18th, Chupra and Ghazeepore; in the latter part Moozufferpore. October, Baulea; 15th, Berhampore and Rungpore. It is only necessary to cast one's eye over the map to perceive immediately how irreconcileable these dates, and the intermediate distances are with the suspicion of local origin."

☞ Compare this with the irruptions of the epidemic almost simultaneously in France, from Lisle to Toulouse!—*Ed.*

Thus it is clear to demonstration that the disease did not originate in Jessore; on the contrary, there is as good, indeed better, reason to suppose that it was carried to Jessore, than that it *first* broke out there. In truth, there is no proof that it sprung up in any one town, or even district, but, from some causes of which we are and ever shall be entirely ignorant, it was generated in the province of Bengal, in several places at the same time, and very probably under similar circumstances. Let what will have been its origin, it did *not* commence at Jessore, nor do we know at what place it did commence, and, consequently, any argument or train of reasoning founded on such assumption is utterly baseless. It is curious, and it should be known, that the epidemic did not, in the first instance, display many of those peculiarities which have since distinguished it. Whilst confined to the province of Bengal, it at once raged simultaneously in various and remote quarters, displaying no predilection for particular tracts, observing no regularity of succession in the chain of its operations. Whilst there existed the same violence in its commencement and rapidity in its progress which subsequently marked it, there were wanting the earliness of declension and entire subsidence, afterwards observed. It rarely altogether disappeared from a town or a tract, but hovered in the vicinity, as though unsatiated yet, and waiting for fresh victims. Thus we see that, bad as the pestilence has been in Europe, it is yet considerably mitigated from its pristine virulence. If it be true, that when once it has set its foot in a region, it continues to spring up in it occasionally, and thus may be considered an addition to our standing army of diseases; still it has lost that character of enduring ferocity which it seemed at first to assume in Bengal. When it has made the tour of the world, it may gradually go out like a flickering lamp, or dwindle into a malady of only ordinary severity. Such has been the case respecting the various plagues which traversed Europe in the middle ages; such has probably been the case, in a still more striking degree, with other pestilential distempers. But this is a matter of speculation; it is certain that, after the present epidemic had become diffused, it observed certain phases or stages of advance, maturity, and decline, which have added to its singularity, but materially diminished its destructiveness.

The only places attacked beyond Bengal, on the eastern side of the Ganges, in the Autumn of 1817, were Moozufferpore, Chupra, and the cantonment of Ghazeepore.

"And now the Epidemic began to shew one of the most striking peculiarities which characterised its march. It no longer pushed its influence, without distinction or apparent choice in all directions, and throughout every tract coming in its way. It began to affect particular lines, and to fix itself in particular divisions of Country; wholly restricting itself for the time to the course of those lines and divisions. Instead of shooting up from Moozufferpore, Chupra, and Ghazeepore, through the contiguous districts of Gorruckpore and Jiounpore, to the provinces of Oude and Rohilkund, it wholly left that part of the Country; and for many months confined itself to the tracts lying west of the Ganges and Jumna. Thus, from the beginning of November, when it quitted Moozufferpore, until the end of March, when it broke out in Allahabad, on the junction of the Ganges and Jumna, it does not appear, that any one spot of the immense tract stretching to the East of these rivers from the northern point of Saharunpore to the Southern boundary of Tirhoot, was visited by the disease. It will be afterwards seen, that from Allahabad, a new stream of the pestilential virus, now apparently propagated by regular succession, issued in various directions, and made a great part of this tract suffer for its previous immunity." 11.

The epidemic did no great mischief, till the end of the first week in November, when it reached the centre division of the grand army, encamped under the personal command of the Marquess of Hastings, near the banks of the Sinde in Bundlekund. On referring to the map we perceive that the Sinde, which is not the great Sinde or Indus, down which the admiral of the Macedonian Conqueror sailed, runs to the north-east to empty itself into the Jumnah, and that nearly parallel to, and at no great distance from it, are three other rivers all taking the same course. Such a locality, we may imagine, must have instantly been marked by cholera as its own; and unfortunately in that place and that time, an army was gathered together, to be more than decimated by that cruel scourge. The whole occurrence is of so interesting a description that we cannot refrain from laying it before our readers in the words of Mr. Jameson, who relates it in a manner equally eloquent and feeling. We do this the more willingly, as only a few lame and mutilated versions have been made current by the modern fry of cholera compilers.

"It was here that the disease put forth all its strength, and assumed its most deadly and appalling form. It is uncertain whether it made its first approaches on the 6th, the 7th, or the 8th of the month. After creeping about, however, in its wonted insidious manner, for several days among the lower classes of the Camp followers; it, as it were in an instant, gained fresh vigour, and at once burst forth with irresistible violence in every direction. Unsubjected to the laws of contact and proximity of situation, which had been observed to mark, and retard the course of other pestilences, it surpassed the plague in the width of its range; and outstripped the most fatal diseases, hitherto known, in the destructive rapidity of its progress. Previously to the 14th, it had overspread every part of the Camp; sparing neither sex nor age in the undistinguishing virulence of its attacks. The old and the young, the European and the Native, fighting men and Camp followers, were alike subject to its visits; and all equally sunk in a few hours under its most powerful grasp. From the 14th to the 20th or 22d, the mortality had become so general, as to depress the stoutest spirits. The sick were already so numerous, and still pouring in so quickly from every quarter, that the medical men, although night and day at their posts, were no longer able to administer to their necessities. The whole camp then put on the appearance of a hospital. The noise and bustle almost inseparable from the intercourse of large bodies of people, had nearly subsided. Nothing was to be seen, but individuals anxiously hurrying from one division of the Camp to another, to inquire after the fate of their dead or dying companions; and melancholy groups of Natives bearing the biers of their departed relatives to the river. At length, even this consolation was denied to them; for the mortality latterly became so great, that there was neither time nor hands to carry off the bodies; which were then thrown into the neighbouring ravines, or hastily committed to the earth, on the spots in which they had expired, and even round the walls of the Officers' tents. All business had given way to solicitude for the suffering. Not a smile could be discerned, nor a sound heard, except the groans of the dying, and the wailing over the dead. Throughout the night especially, a gloomy silence, interrupted only by the well known dreadful sounds of poor wretches labouring under the distinguishing symptoms of the disease, universally prevailed.*—The Natives thinking that their only safety lay in flight, had

* "Many of the sick died before reaching the hospitals; and even their now begun to desert in great numbers; and the highways and fields for many miles round, were strewed with the bodies of those, who had left Camp with the disease upon them, and speedily sunk under its exhausting effects. It was clear, that such a frightful state of things could not last long; and that unless some immediate check were given to the disorder, it must soon depopulate the Camp. It was, therefore, wisely determined by the Commander in Chief, to move in search of a healthier soil, and of purer air. The Division, accordingly, on the 13th marched in the South Easterly direction towards Talgong, and Sileia; and after several intermediate halts, on the 19th crossed the clear stream of the Betwah, and upon its high and dry banks at Erich, soon got rid of the pestilence, and met with returning health. But its line of march, during the whole of this progressive movement, exhibited a most deplorable spectacle. Although every means had been taken, by giving up the ammunition carts, and collecting elephants and draught cattle, to procure sufficient carriage, the sick were found too numerous to be moved, and were in part necessarily left behind. And as many who left the carts, pressed by the sudden calls of the disease, were unable to rise again; and hundreds dropt down during every subsequent day's

---

comrades whilst bearing them from the out-posts to Medical aid, sunk themselves, suddenly seized by the disorder.—Never was the impressive language of Scripture more applicable than now:—'*In the midst of life we are in death.*'—All security of life was gone; and as youth and vigour afforded no safety, even the healthiest man could not in the morning tell, that he might not be a corpse before night.—Such was the dreadful effect of the scene, that even long after its occurrence, it could hardly be described without shuddering by the eye-witnesses.—How fatal the sickness of this Division, had it continued much longer, might have proved in its political consequences, we have been told from the highest authority. In delineating the rise and progress of the late war, on his return to the Presidency, the Governor General thus spoke of the visitation of his Army.—'The dreadful pestilence, which made such havoc in the Division under my immediate command, forced me to quit the banks of the Sinde, and to seek a more favourable country for the recovery of my numerous sick. I did not find this until I was fifty miles from the river which I quitted.—Fortunately the change of air was rapidly beneficial; for, a very short time had passed when I received intelligence of an invitation said to have been given by Scindia to the Pindarries.—He was reported to have promised them, that if they would come so near to Gwalior, as to make his getting to them easy, he would break his Treaty, and join them with the Force, which he had at his capital.—The Pindarries were in full march for Gwalior, without meeting even a shew of impediment from the troops of Scindia stationed in their route; though the co-operation of his army for the extinction of the Pindarries was an Article of the Treaty.—We hurried back to the Sinde; but this time we chose a position nearer to Gwalior, than what we had before occupied.—We were within thirty miles of the City, and our advanced guard was sent to occupy the passes through the hills which run at some distance South of Gwalior from the Sinde to the Chumbul.—Those passes were the only route by which communication could take place between the Pindarries and Scindia: and I was nearer to support my advanced guard than the Maharajah was to attack it, could he bring his mind to so desperate a stake.—The Pindarries finding their hopes baffled and the passage stopped, attempted to retire; but they had been followed close by our Divisions, were surprised, dispersed, and slaughtered in a number of small actions. In short they disappeared. And thus our objects were completed.'—A few days' longer continuance of the Epidemic might, by entirely crippling this Division, have given a very different appearance to the face of affairs, and prolonged the struggle to an indefinite period."

advance, and covered the roads with dead and dying; the ground of encampment, and line of march, presented the appearance of a field of battle, and of the track of an army retreating under every circumstance of discomfiture and distress. The exact amount of mortality during these few calamitous days could not, from the circumstances of confusion and general disorder, under which it took place, be ascertained with any degree of accuracy. From the Military returns however it appears, that in this fatal week, of 11,500 fighting men of all descriptions,* 764 fell victims to the disorder; and of the camp followers, it was conjectured, that about 8,000, or one-tenth of the whole, was cut off." 17.

On this narrative we may make a few remarks. The disproportion between the numbers of troops and of camp followers who fell, is very remarkable; of the latter one-tenth, of the former one-fifteenth. Yet this disproportion is trifling in comparison with what has since been observed. The camp followers, ill fed, ill provided for in every way, have invariably suffered more than the better circumstanced troops. This might be expected. In the next place, we doubt whether so much should be attributed to the change of quarters on the part of the grand army, as is usually done. The epidemic commenced on the 6th or thereabouts, and declined on the 22d or 23d, having raged for fifteen or sixteen days. Subsequent events have shewn that in India it never continued in vigour in a camp for a longer period than ten or fifteen days. Whether this be or be not the whole truth, we think that it ought at least to be taken into consideration, in reasoning on the event. We have frequently made this reflection on perusing similar relations. Mr. Jameson, we find, is struck with the same idea, which tends to give it additional weight.

We will not pursue the evolution of the epidemic throughout the province of Bengal, nor give a dry detail of names which would convey no information to ninety-nine readers out of the hundred, and of circumstances devoid of any general utility. We shall merely notice events which may be striking or singular in themselves.

Whilst the inhabitants of Hutta and Saugor and the dependencies of Nursingha and Puthooreea suffered very severely, "the troops serving in that quarter, and some of them living in the very centre of the pestilence at that time, scarcely appeared subject to its influence." In June it appeared at Kotah, where it is said to have cut off a hundred men a day, but having got into the high and mountainous track it gradually died away, and never reached the states of Oodeypoor and Ajmeer. To shew the great inequality of the disease in severity, we may mention what occurred to the troops under Major-General Marshall. During their march they fell in with the disease at Jubbulpore on the 9th of April, and suffered during the remainder of the month. Of 8,500 fighting men, only 125 were taken ill, and 49 died, or a fraction less than one in 173.

Nagpore and Mooltay were both severely visited, yet in the intermediate space of 70 miles the disease was not met with, and Baitool, a large town

---

* "The number of fighting men in Camp is here a good deal overrated; for one Detachment left Camp before the commencement of the disorder; and another much larger as early as the 18th.—The two together amounted to nearly three thousand."

in the direct road from the river to Mooltay, was entirely exempt. At Nagpore, the Bengal Reports from the West are terminated. From Nagpore it passed to Jaulnah and to Bombay; from the latter Presidency a distinct Report has proceeded, and been already analyzed.

"The Nagpore Subsidiary Force under Colonel Adams, afforded the first striking instance of a large body of men coming into the pestilential medium, and from the previous enjoyment of perfect health, falling at once into a wretched state of sickliness. This Division had, during the early part of May, been occupied in besieging the important Fortress of Chanda; but, although it had undergone excessive fatigue, and met with a few casualties from constant exposure to the sun, it had nothing like marked disease, until, on the morning of its last march on the return to Nagpore, it encamped at Gaongong, a village situated nine miles South of the city. Here it had hardly learnt that the epidemic was raging in the vicinity, when it began itself to experience its unwelcome visits. As usual, its first assaults were most severe. Many of those attacked whilst loitering for water in the neighbouring rivulets, were brought in expiring; some dead. Of 70 cases admitted during that night and the succeeding day, about 20 died.—On the 31st the instances of attack were equally numerous; but in these, the exhaustion was not so sudden, and the subsequent symptoms were less severe. On the 1st of June, the Division moved from Nagpore towards the cantonments at Hoshungabad. The disease then gradually declined; and almost entirely disappeared on the 17th and 18th, after some seasonable falls of rain. The Madras Troops, composing part of the besieging force employed at Chandah, are reported to have suffered equally with their comrades from this side of India." 23.

We have hitherto accompanied the epidemic to the West and to the South. We have not individualized its successive evolutions, a dry and tedious process; we have merely directed attention to such as were remarkable. We shall do the same, as we follow the monster in his march, or rather his irruptions, on the North and East. The point from which we start is the junction of the Ganges and Jumna at Allahabad, where the disease established itself in the Spring of 1818, prevailing for several months with great malignancy, and sweeping off in the town and district nearly 10,000 persons. The troops stationed in the fort and city were not affected until the middle of July following, although holding daily and unrestrained intercourse with the towns' people. In the following passages we shall see that the disease, in India as in Europe, shewed itself most virulent and most intractable in filthy and crowded communities. We shall also see how frequently it passed from one point to another at a considerable distance from the first, but forbore to affect the intermediate towns or villages.

"On the Jumna, *the epidemic spared Culpee, and almost every intermediate village between it and Etawah.*—The latter place it reached late in May; and, after doing considerable mischief, it would appear to have thence at once stretched across the Doab to Futtigur, visiting very few places on its route. It appeared in the lines of Futtigur on the 10th of June; and thence was communicated to the town. There it shewed little virulence of symptoms; and wholly subsided on the commencement of the rains early in July. It is curious, that Muttra, situated considerably higher up the Jumna than Agra, should have had the disease in the beginning of June, whilst the latter place was not visited until the 1st of July. *In Muttra, a filthy and crowded city, the disease was very virulent, and the mortality great.* In Agra, a dry and airy town, the symptoms were mild and the deaths few. The Cantonments attached to Agra remained nearly exempt; but those at Muttra being low and near the banks of the river, partook of the general unhealthiness of the town, and lost many men. In both places, the epidemic loitered for more than a month. It then, on the 11th of July, entered the town of Coel; which was alone affected in the Alligur district: the Jails, Cantonments, and the adjoining villages enjoying perfect immunity. We next find the disease on the 20th at Delhi. Here it remained nearly a month, and committed very considerable havoc among the condensed population of an extensive city.

Neither between Agra and Delhi, nor during its route from the latter place to Meerut, did the disorder halt in any of the intermediate towns and villages. Since, of these many were placed very low, and much exposed to masses of animal and vegetable putrefaction, and every other source of miasmata and contagion, their immunity from attack could hardly be ascribed with justice to peculiar healthiness of situation. The reason rather seems to have been that, in this quarter, the epidemic, whether from the pure and elastic air of the Northern Provinces being less favourable to its existence, than the thick damp climate of Bengal; or from its beginning to give way to that general law of Nature, which requires that diseases, like all other things, should have their decrements as well as their rise and increase; or from some other hidden agency altogether unsuspected, was now beginning to die away, and could only be kept alive by strongly exiting causes: such as large bodies of men crowded together in camps and cities. Hence, we find, that between Delhi and Meerut,—a distance of thirty miles, populous and studded with considerable towns and villages,—not a case of the disease occurred;* whilst the latter city, and the cantonments attached to it, suffered under it from the 28th of July to the 20th of August. Few were seized in the town, and fewer in the lines; and in both the sum of mortality fell considerably short of three hundred. So likewise, between Meerut and Saharunpoor, the epidemic kept dormant; whilst the latter city, which is very low and filthy, filled with ruined buildings, and intersected by foul channels with oozy banks, suffered considerably. The disease shewed itself in the town about the end of September; was in full force about the middle of October; and declined from that time until the last week of the month, when it wholly disappeared. It may be, however, that the virus proceeded not in a centrical line from Delhi to Saharunpore, but along the course of the Jumna; as Tannah, a large town, only sixteen miles from the river, and twenty South of Saharunpore, was attacked before the latter place." 28.

The epidemic can be traced no further North than Saharunpore, the high mountain ridges checking it, though the poverty and wretchedness of the inhabitants seemed to render them equally likely to take it, and to suffer severely from it when taken. How perplexing the rationale of the outbreak of the disease in detachments of military has ever been in India, we might readily prove. It seems clear enough that such detachments passing through places where the disease existed, were frequently affected shortly afterwards. This looks like the sequence of effect and cause, and probably it was so. But granting this, which we readily do, it is obvious that the influence of contagion is not demonstrated by such examples; on the contrary we think that the circumstance is rather adverse than favourable to the idea of such agency. It is passing strange that a body of troops marching round, or by, or even through a place where an infectious disease ex-

* "By a subsequent report it indeed appears, that the epidemic slightly visited the towns of Ghazeeabad and Mooradnuggur; but in them the cases were so few as hardly to affect the statement in the text."

ists, should happen to come in contact with those who have it, or with those who can convey it. Suppose that a very contagious malady, an exanthema, for instance, measles, were prevailing in London, and that a body of king's troops were to pass through the metropolis on their way to put down a Reform insurrection in the North; should we probably find those troops become affected, should we look for their becoming so? We should not, and of many divisions so circumstanced we should certainly expect that the majority would escape. But if the prevailing disease were another sort of epidemic, an ague, for instance, our expectations would be widely different, and probably no body of men passing through the metropolis at such a time would fail to be attacked by it. This is merely an argument founded on probabilities, yet we think that it shews the impossibility of proving or even supporting the doctrine of contagion by such instances.

But this is not all. If the detachment affected by passing through a district in which cholera is raging, communicate the disease to another detachment previously healthy, that is, if the latter are attacked by cholera very shortly after coming into contact with the former, shall we not be forced to acknowledge the agency of contagion or infection on this occasion? This will not admit of a direct reply. As only a very few instances of this kind were adduced we deem them inconclusive—first, because the disease was so irregular in its progress, in its appearance, and its disappearance, that the occurrence might have been a mere coincidence; secondly, because the observation of the fact itself might have been incorrect. This is no excessive fastidiousness on our part; it is the mere application of the ordinary cautions in the admission of evidence to this particular case, the mere illustration of the precept, post hoc non ergo propter hoc.

If, indeed, such examples were numerous, common sense and rules of reasoning equally demand the abandonment of these scruples. If one thing follows another, in the majority of cases that admit of their sequence, we are bound to admit them as cause and effect, in the absence of positive proof, or very satisfactory arguments to the contrary. It is equally unphilosophic to reject a general rule because it has exceptions, and to erect an exception into a general rule. In point of fact, the cases of communication of cholera from one detachment to another are neither numerous nor satisfactorily authenticated. The following, for instance, may be considered as rather a favourable sample of the whole; yet in this we shall find that, previously to the arrival of the body which is accused of bringing the cholera, some cases had occurred amongst those who are said to have received it. It must be strangely constituted minds, indeed, that can feed on such a dish as this.

"On the 23d of July, a Detachment, consisting of a troop of the Horse Artillery Brigade, the Rocket troop, and 5 Companies of the 1st Battalion 25th Native Infantry, marched from Meerut to join the force collecting at Hansi under Brigadier Arnold, for service in the Bickaneer and Bhatty countries. They remained perfectly healthy on their march to the Jumna, and during several subsequent days, in which they were encamped on its East bank. On the 29th they crossed the River, passed through Delhi (the disease being then at its height in the town) and encamped outside of its walls, about a mile to the West. On the 30th, they continued their March in a North-westerly direction; and on the morning of the 31st, they were attacked by the Epidemic.—It continued unabated amongst them, until the 6th; when they joined the general camp at Hansi. *Although one or two cases had occurred in this Force a day or two pre-*

*viously to the arrival of the Meerut Detachment*, it was the unanimous belief of the Medical Staff serving with it, that from the latter, it got the disease in its epidemical form. What gives a colour of probability to this conjecture is, that the disorder was not met with in any of the villages lying in the immediate line of the route of the Detachment from Delhi to Hansi; and certainly did not appear in the latter town until some time after its prevalence in Camp, and then but very slightly.—On the other hand, it was generally rumoured that, several intermediate places between Delhi and Hansi, and betweeen Delhi and Kurnaul, particularly Paniput, had been affected previously to Hansi being brought within the pestilential influence.* It continued with the troops until the 12th of August; and accompanied them in their march in a W. N. W. direction to Futtihabad, Rhauneea and Sirseea, and in their retrogade movement on Hissar. Of the whole Force, only about 250 men were attacked. The symptoms were comparatively mild; and the deaths few. The Epidemic did not reach Loodeehana; and appears in this quarter to have been limited by the river Sutledge.

From Delhi or Aligurh, the disease would seem to have spread in a South West direction to the principality of Jeypore; the capital of which it reached in the latter end of August. Here it was neither malignant nor general: its attacks being almost confined to the most wretched class of the inhabitants, and the whole mortality throughout the circumjacent country scarcely exceeding a thousand men.—On the 12th September it began to abate in the city; and on the 14th it entered the camp of a detached Force commanded by Major Agnew, at Titirya, twenty-five miles South West of Jeypore; and there raged with very considerable virulence till the 28th, when it gradually abated. Of 96 Europeans, and 4100 Natives composing the fighting part of this Force, the admissions were 292, of whom 122 died. The mortality amongst the camp followers could not rightly be ascertained. The Epidemic was no more heard of in this direction: neither the town, nor the valley of Ajmeer being affected; although another large division of the Rajpootana Force was encamped in the latter, on similar ground, and under circumstances apparently in no wise differing from those of their less fortunate comrades at Titirya." 31.

In the middle provinces of Bengal, it maintained its character for eccentricity, decimating one place, totally omitting another, merely playing with a third. It is curious that the troops and camp followers in personal attendance upon the Marquess of Hastings, on his return from the upper provinces, again fell in with the epidemic on the 20th or 21st of April; but now its attacks were nearly restricted to such persons as had not been with the centre division of the army in the preceding Autumn.

"In the foregoing pages, the singular fact of the virus shewing an unwillingness to ascend high and mountainous tracts of Country, has been noticed.—Thus it wholly avoided Kumaoon, the Hilly Districts North of Hurdwar, and the elevated stony belt which girds in the Rajpootana States to the North West.—But this rule was not without exception; for, in June, it passed the lofty range of mountains guarding Napaul to the East, and visited Khatmandoo, Patun, and Bhatgoon in the subjacent valley; and, in October following, it got from Sylhet to the independent countries of Kashar and Munnipore, on the eastern borders of Bengal. But, even here, it might be seen that high lands were not congenial to it; for it had been raging with very great violence in the adjoining District of Sylhet, before it was enabled to overcome the obstacles opposed to its progress

---

* Had a detachment of soldiers, with some cases of cholera, arrived in Haddington from Newcastle, a few days before the disease broke out in the former, would we ever have heard the last of such a proof of contagion?

by the intervening mountains.—The same causes may be assigned for its partial occurrence among the Ramgur and Sirgooja Hills; whilst Sonepoor and Sumbhulpore were largely affected: apparently from Cuttack, along the course of the Mahanuddee." 33.

Why the disease should stop at a mountain range, supposing it essentially propagated by contagion, we know not. It may be said that there is little or no communication over mountains. This has never been proved, and, in the absence of positive evidence, is more likely to be false than true. In India, when a place was smartly assaulted by the epidemic, the inhabitants almost invariably fled into the villages and country places; they had no belief in its contagiousness, and, consequently, no obstacle was opposed to their dispersion, no objections offered by the inhabitants of the open country, or of neighbouring towns, to their reception. If the epidemic visited the plains at the foot of mountain ridges, it is more than probable that the panic-stricken people would flee into those mountains or cross them for safety; and when once the comparative salubrity of hilly districts was established, such emigration would be likely to be much increased. These are probabilities at all events, and may counterbalance any vague and general assertions on the part of the contagionists. The ensuing quotation, on the duration of the epidemic in different parts of Bengal, will conclude our notice of its origin and progressive evolution in that Presidency.

"As no more favourable opportunity will hereafter occur, a few particulars may be here mentioned regarding the times of appearance and periods of duration of the Epidemic during its stay in Bengal.—It has been stated in the text, that it first broke out epidemically in Calcutta in August 1817; and that it continued prevailing in the city, with more or less violence, till the ensuing August. From that time until the following April, when it recurred with considerable violence on occasion of some variable, unseasonable weather, only a few cases came to notice. Now (May) it has again subsided; but fresh instances of the malady are sure to occur, as a consequence of the sudden vicissitudes in the atmospherical heat and moisture, produced by every North Wester.—It has probably observed a like course in Jessore; but its progress there cannot be exactly learnt, in consequence of the absence of a Medical officer from that station for many months.—It appears, however, that since the beginning of the present year it has been constantly in existence, and destroying many persons in the North and Eastern parts of the district.—In the Backergunge district it remained until the end of last year, since which time it would not appear to have done much injury. —In Bullooah and the tracts near the mouth of the Ganges, it began in February, and terminated in June, 1818.—In different parts of Mymensing it has existed since June, 1817. So likewise in the city and District of Dacca.—In Tipperah, after wholly disappearing during the Cold Season, it returned in March, 1818; and remained till the breaking up of the subsequent Rains, from which time it seems again to have retired.—In Sylhet its career has been singularly various. After retiring in October, 1817, and being for several months dormant, it returned in the end of March, and subsisted till the setting in of the Rains, when it a second time nearly disappeared. But, about the middle of October, it suddenly increased all over the district to as great a degree, and with greater fatality, than on its first appearance. It gradually declined in December, and again withdrew in the end of the year. Finally, it made its fourth visit in the commencement of the present Hot Weather; but not generally.—In Rajshahy, it prevailed most in September and October; and disappeared about the middle of November, 1817. It again appeared in the following Hot Weather, and frequently proved fatal.—It commenced in August in the city of Mooshedabad, and has continued till now; but always in a very mild shape.—In Nuddeea, as we have seen, it began generally in July and August. Towards the latter end of the year, it became less severe. With the heavy rains, which set in towards the end of the following February, it recurred in various parts of the District. As in Calcutta, it declined in August following, was very rare in the cold months, and again got head in the beginning of the present Hot Weather.—In Burdwan a few cases happened early in the Rains. In September the mortality caused by it was alarming; and, from that month until the end of the following year, few days passed without the disease appearing in some of the villages. It was particularly violent in the commencement and at the termination of the Hot Winds. Since January, 1819, no account of its progress in that quarter has been received.—In the district of Bhaugulpore it continued its ravages, without intermission, from August 1817, to May 1818, when it considerably abated; and from that time till now only occasional cases have occurred.—At Monghyr it began in the latter end of August; decreased in October and November; was not heard of in December; recurred partially in January and February; increased much throughout the Hot Weather; and finally altogether withdrew in September. In Behar it began in August; nearly disappeared in the Cold Season; recurred about the 20th of February; and continued active till the ensuing November; when it again nearly ceased; to re-appear in March and April. Of late the disorder has appeared much in the South West parts of Bengal, visiting severely Midnapore and Cuttack, which it almost spared in the early part of its course. —But it must not be supposed from the foregoing statement, that the disease now rages with that width of range, or generality of attack, which it took in the first year of its existence. In Calcutta certainly, and probably in almost every other part of the Lower Provinces, it is no longer strictly Epidemic. The case is this. A disposition to produce the disorder still exists in the atmosphere. This is brought into play by sudden alterations of the weather. Then perhaps a dozen of cases, and two or three deaths occur; the Settlement is immediately thrown into alarm; and it is generally rumoured, that the cholera is more prevalent than ever. The truth nevertheless is, that more individuals probably perished from the disease in one day in the Autumn of 1817, than now do in a quarter of a year. It should, moreover, be recollected, that the disease is endemical in Bengal in the sultry periods of the year; and that were it not for the present scared state of the public mind, the few cases now occurring might, as in former years, pass nearly unnoticed. As it is, all is magnified by apprehension. As is quaintly observed by one of the Medical Staff, 'the disease, like some foul fiend is still wandering to and fro, and like the author of all mischief spreads universal terror before it.'—The disorder having in April severely attacked several detachments of troops marching through the Cuttack and Midnapore districts, a notion was entertained, that there was something in the vicinity of the Soobanreeka river, which divides these tracts near Balasore, connected with, and immediately leading to its rise. But this was quite a mistake; for it was learnt upon enquiry, that the several divisions got the disorder equally readily in different parts of Cuttack; and that it always broke out after a fall of rain, or other manifestly strong exciting cause. Besides, that the epidemic has been long known at Balasore, and had visited several detachments in the Cuttack jungles so far back as the Cold Weather, and Juggurnath lines in the Spring of the preceding year." 37.

A retrospect of the origin and progress of the epidemic in Bengal would give us much trouble, and our readers little amusement. Those who like may do it for themselves. It is enough to say that it evidently arose about May or June, 1817, after seasons remarkable for irregularity, and a preceding year distinguished for epidemic sickness and mortality. It did not spring up in Jessore, nor is it proved to have originated in any one town or even district. But it was first observed in the plains of Bengal in the neighbourhood of the Ganges. Near a sacred river it was born, and for rivers it has ever subseqently testified a strong regard. What its causes were we know not, we shall never know; but if, as the evidence distinctly proves, it originated at once in distant districts, we must necessarily conclude that those causes were independent of each other. From its native places it diverged in all directions, but its tendency to the West and South was almost as marked at the first, as it has since remained. In its progress it displayed singularities and excentricities which have rendered it proverbial, but these were always shewn in the detail; its progressive evolution was westward. In the record of its atrocities we can still decipher, though sometimes darkly, that the needy and the debilitated were most frequently its victims. Yet the cholera of India, as of England, would seem to have disdained all laws, even its own; and, as if to vindicate its power and confound those who were inclined to limit its influence, it occasionally burst with fury on an army, a town, or a district, sweeping off victims of all descriptions, and attacking classes which it usually spared. Even here, however, we may pick out something like a system, and whenever communities or masses suffered greatly, we may commonly perceive, on close inspection, that they were weakened and predisposed by some efficient agency.

### SYMPTOMS OF THE DISEASE.

We need not, and of course we shall not, go at length into these; they are as familiar as household words. But it will be necessary to advert to some particular points. It has been roundly asserted that the diarrhœa which has generally been found to precede the attack of cholera in Europe, and the fever which has usually been observed to follow it in those instances where the patient was not cut off early, were noticed and described in the Indian epidemic. In our analysis of the Reports from Bombay and Madras, we have shewn that this assertion is untrue, as regards the disease in those parts of Indostan. Let us see what was the case in Bengal.

"The attack was generally ushered in by a feeling of fulness and pain in the stomach, and swelling of the abdomen; with sickness, and a desire to go to stool. Then came, *almost immediately, vomiting and purging of a pale thin fluid,* without taste or smell; great anxiety; oppression, and sense of constriction about the heart and præcordia; thirst and internal heat. These symptoms were accompanied, or quickly followed, by severe cramps; generally beginning in the fingers and toes, and thence extending to the wrists and fore-arms, calves of the legs, thighs, abdomen and lower part of the thorax.

Together with these signs of general depression, the action of the heart and arteries was uniformly diminished. The pulse sunk rapidly at the wrists and temples; and at last could no longer be felt, or was merely perceptible by a slight and indistinct fluttering. The respiration became laborious and hurried, with sighing, and long and frequently broken inspirations. As the blood forsook the extreme vessels, and withdrew to the great cavities, the exterior surface of the body grew pale, shrunken, and cold. The skin became clammy, dank and disagreeable to the feel; bedewed with large drops of cold sweat; and discoloured of a leaden, bluish, purple, or livid hue. The countenance was greatly changed; the features were contracted, collapsed, and ghastly.—The eyes sunk in their sockets; fixed and glassy; covered with a thick film; heavy, dull, suffused, and surrounded by dark brown or black circles.—The lips livid, or of a purple colour.—The finger-nails blue; the palms of the hands white, bleached, and puckered into folds.—The mouth was dry and parched; the tongue bluish, or white and faltering; and the voice hoarse and low.

There was sudden and great prostration of strength.—The hands trembled; and the action of the voluntary muscles was uncertain and unsteady.—The patient could no longer stand or walk without assistance: He became as feeble as a child; staggered like a drunken man; and unless supported, sunk down like one in the last stage of debility from fever.

In feeble habits, and where the disease attacked in extreme violence, the scene was soon closed.—The circulation and animal heat were never restored.—The spasms, vomiting, and purging were frequently renewed.—The thirst continued incessant and unquenchable; and was no sooner gratified by draughts of water, or other fluid, than dreadful retching ensued.—The burning heat, anguish, agitation, and restlessness continued unabated. At length, the patient, exhausted by the depressing influence of the malady, and the repeated large discharges, fell into a listless state; and had no longer strength for either full vomiting or purging. A little fluid was now only rejected by the mouth, as the abdominal muscles were thrown into spasm; or passed off involuntarily downwards, as the body turned round in bed.—The patient remained deadly cold; grew weaker and weaker; and insensibly sunk into death; or was carried off during a repetition of spasms; sometimes in one, but more frequently within four, six, or twelve hours." 40.

After adverting to the fatality of the collapse, and the unquenched and unquenchable thirst, Mr. Jameson proceeds thus.

"Much variety, however, occurred in the kind, order, and sequence of the symptoms; according as the virus of the disease happened to be more or less concentrated, or the individual affected of a strong or feeble constitution. *Vomiting was the symptom of earliest and most frequent occurrence.* Next came purging; then the cramps, and spasms. Frequently, however, this order was reversed, and the purging and spasms took the lead of the vomiting. Sometimes the cramp preceded both. Sometimes there was no vomiting; sometimes no purging; sometimes no spasm, throughout. Sometimes all these symptoms were simultaneous; and the vomiting and purging took place together, as if caused by sudden contraction of the alimentary canal in its whole extent. In some rare instances, the virulence of the disease was so powerful as to prove immediately destructive of life; as if the circulation were at once arrested, and the vital powers wholly overwhelmed. In these cases, the patient fell down, as if struck by lightning; and instantly expired. Others, again, sunk after making one or two feeble efforts to vomit, and drawing a long and anxious inspiration. Some recovered from the insensibility produced by the first shock, and afterwards went through the regular course of the disease." 42.

The foregoing passages set at rest the question of antecedent diarrhœa in the cholera of Bengal. Comment upon them is superfluous; and if any one is hardy enough to affirm that in this particular the European and Indian epidemic have not displayed a striking *general* difference, the statement is only to be met by a flat denial, and a confident reference to the only authentic and trust-worthy documents we possess. Argument there need be none; it is a matter of evidence, and of strict deduction from evidence only.

Passing over particular symptoms, which we do for want of space not inclination, we arrive at the consideration of the more immediate consequences of the attack; in fact, at that of consecutive fever. If our readers have referred to the Bombay and Madras Reports, or to our reviews of them, they will find that the former expressly declares that fever was not observed in that part of India, whilst the latter implies nearly the same thing. It has been imagined that consecutive fever was as common a consequence of cholera in Bengal as in Europe. This will be found to be an error. It appears that Mr. Jukes, an individual reporter, did mention in his letter the occurrence of secondary fever, yet that, if the description be carefully considered, was not only not identical, but indeed hardly similar to, the form which we have seen in this country. The present report, which may be looked on as the sum, or co-efficient, of the experience or expressions of many, is rather vague in this particular. We shall lay before our readers all the information which it offers on the point.

" Among the Natives generally, where the attack was exceedingly severe, the constitution sunk with scarce an attempt to rally; and of those who recovered, the secondary stage was of short duration, and unaccompanied with much reaction. In the milder cases, the attack either was repelled by the unaided powers of life itself, or readily gave way to the simplest means of cure. The pathognomonic symptoms of the disease speedily abated; the patient sunk into a profound and quiet sleep; and the breaking out of a warm equable perpiration all over the body, evinced the restoration of the vital powers, and might be considered an almost infallible sign of recovery. *In such cases, slight debility, and irregular action of the intestinal canal, were the only ill consequences of the attack;* and a copious discharge of bile, or fæculent matter, either natural or procured by the exhibition of a single dose of a simple purgative, completed the cure." 51.

We should observe for the sake of accuracy and clearness, that "the secondary stage," above alluded to, was not the stage of fever, but of mere reaction. This is distinctly pointed out in a preceding passage, which we need not quote, as the context is of itself sufficient to explain it. In the milder cases, then, among the natives, we see that there was really no consecutive fever, a feculent or bilious stool completing the cure. Has it been so with "the milder cases" in Europe? As a general rule, we say that it has not. " But hold," cry the importationists, " why not tell us what occurred in the severer cases? In them it was that the distinctive fever occurred; by them the identity we insist upon is proved. It is true that we cannot procure evidence of antecedent diarrhœa; it is true that we cannot convince you, obstinate as you are, and peculiar as the construction of your minds must be, of the existence of fever in Bombay and Madras, in opposition to the written statements of those Boards; all this we regret to say is too true, but all this amounts to nothing, if we can demonstrate by the shadow of a shade of proof, that consecutive fever was observed in Bengal. We do not care for the particular kind of fever, we do not mind any little difference it may display from its European progeny or rather self; all we want is fever; and if by any means we can procure it, you may rest assured that we will make the most of it." Well, then, we will satisfy the anxiety of these gentlemen.

" But, in the more violent forms of the disease, recovery was longer protracted; and the sufferings of the patient were more severe.—After the most distressing symptoms had been in great measure subdued, he was still harassed by constant thirst, irritability of stomach, pain and soreness of the Epigastric region, watchfulness, and confused dreams. The stomach and bowels did not for a long time regain their usual tone; and the frequent occurrence of *obstinate dysentery or diarrhœa proved, that almost irreparable mischief had been done to the whole of the chylopœtic viscera.* In these cases the debility was great, and of long duration; and the strictest attention was required during many days, to prevent the patient from sinking entirely.—Sometimes, the debility terminated in incurable dropsy.—In some instances, partial loss of vision; in others, of hearing, ensued. In one man paralysis of the bladder and lower extremities occurred early, and continued long after the cessation of the common symptoms of the disease.

It was almost uniformly observed, that health was soonest restored in those cases in which fæculent, black, and acrid motions were early procured; and that on the other hand, their absence was almost uniformly marked by feverishness, sour eructations, flatulence, constipation, and other signs of want of tone and sluggish action of the hepatic system.—*Fevers of the remittent, and intermittent type were among the most frequent sequelæ of the disorder;* but among natives, and especially those of weakly frame, *they could not be considered to form an essential part of the attack.* They were hardly ever immediately superinduced upon the collapsed stage, *and seemed rather an incidental affection, in bodies much predisposed to take on new forms of disease by great existing debility.*

When the disease ran its full course with Europeans, and with Natives of robust athletic make, the following appearances generally presented themselves. What may be termed the cold stage or state of collapse, usually lasted from twenty-four to forty-eight hours; and was seldom of more than three complete days duration. Throughout the first twenty-four hours, nearly all the symptoms of deadly oppression; the cold skin, and oozing of clammy sweat from every pore, the feeble pulse, occasional vomiting, purging, and cramps; the thirst and anguish continued undiminished. Then the system shewed symptoms of revival; the vital powers began to rally; the circulation and heat to be restored; and the spasms, sickness and desire to go to stool, to be considerably lessened. The warmth gradually returned; the pulse rose in strength, and fulness; and then became sharp, and sometimes hard. The tongue got more deeply furred; the thirst continued with less nausea. The stools were no longer like gruel or rice water; they, usually between the third and sixth day, became first brown and watery; then dark green, black, and pitchy; and the bowels during many days continued to discharge immense loads of vitiated bile; until with returning health, the secretions of the liver and other viscera gradually put on a natural appearance. These discharges were generally hot, acrid, and passed with griping and tenesmus. Sometimes they were of a bright yellow colour; and the surcharge of bile was so great, as to be ejected in a pure stream from the stomach. It was remarked that, where the motions consisted of a chocolate-coloured fluid, with floculi swimming in it, the patient rarely recovered.

*The fever, which almost invariably attended this second stage of the disease, may be considered to have been rather the result of an effort in Nature to recover herself from the rude shock, which she had sustained, than as forming any integral and necessary part of the disorder itself. It partook much of the nature of the common bilious attacks of these latitudes.* There was the hot dry skin; the foul, deeply furred, dry tongue; parched mouth; thirst; sick stomach; depraved secretions; restlessness; watchfulness; and quick variable pulse; sometimes with delirium, stupor, and other marked affections of the brain.

Generally, when the disorder proved fatal, after reaching this stage, the tongue, from being cream-coloured, got brown, and sometimes black, hard, and more deeply furred; the teeth and lips were covered with sordes; the state of the skin varied, chills alternating with heats; the pulse became extremely quick, weak, and tremulous; hiccough, catching of the breath, great restlessness, and deep moaning succeeded; and the patient soon sunk, incoherent and insensible, under the debilitating effects of low nervous fever, and frequent dark, tarry, alvine discharges.

In other cases, this secondary period ran a somewhat different course. As the action of the heart and arteries was renewed, and the natural warmth of the body returned, an unusual degree of energy succeeded. The brain was evidently affected; and the patient was quite insensible to the great danger into which he had fallen. The pulse rose as high as 120; great heat, especially over the large cavities, was complained of.—There was extreme agitation and distressing thirst. The patient continually called for cold water, to relieve the burning sensation of the abdomen. Sometimes a warm perspiration broke out near the wrists and forehead, which afforded temporary relief to his sufferings.—To this state of excitement that of collapse quickly succeeded. There was then great prostration of strength; the bowels became quite torpid; severe pains occurred low down in the abdomen, near the site of the rectum, which were always aggravated upon stools being procured by medicine. The state of the stomach now excited surprise; its unnatural irritability was entirely gone, and the most nauseating medicine could be poured into it without exciting vomiting.—It rarely occurred that the patient survived the great sinking produced by this stage; and even where good fortune and the strength of his constitution carried him through it, he suffered long after from debility and disordered bowels." 56.

None who read impartially the foregoing description, can consider it as perfectly applicable to what has been observed in Europe. As a corollary to it, we may mention some facts related by Mr. Jameson. In the centre division of the army, where, it will be recollected, the mortality was so frightful, "it was singular that neither with Europeans nor Natives were those symptoms of strong re-action, and subsequent fever, which among the former at least, were of almost constant occurrence in the lower parts of India, observed." In many the recovery, we are told, was remarkably rapid.

In the Nagpore Division, "the only general after-effect of the attack, was extreme debility." In the Rajpootana Force, "fever was a rare consequence." In Jeypore, "the sufferers generally recovered surprisingly fast; in some instances in a few hours." In the Hansi Division many had to undergo fevers of the remittent or intermittent type. At Delhi, Meerut, Coel, Agra, and Futtigur, "the disease ordinarily left no other ill effects than weakness and disordered bowels."*

So much for the consecutive fever. It will be noticed that the fever that did ensue, was observed in the lower, marshy parts of Bengal; that it assumed a remittent and intermittent type; that it seemed rather a sequela than an integral part of the malady; and, finally, that it was limited, or nearly limited to the severer cases. When the foregoing circumstances are

---

* Those who quote the Bengal Report to shew that the consecutive fever was present in the epidemic there, seem not aware of the dilemma into which they draw themselves in respect to the argument of IDENTITY! We have shewn that the consecutive fever was not observed in the Presidencies of Madras or Bombay. Taking then the admission of the Bengal Report in its fullest latitude, it only proves that the Epidemic on one side of the Jaggernaut Pagoda had consecutive fever, on the other side none! On the left bank of the Kestna fever followed the spasms and purging—on the right bank there was none! On one side of the Ghaut Mountains we had consecutive fever—on the other side nothing of the kind! But the disease having crossed the Indus, the Euphrates, and the Caspian, the epidemic, all at once, resumed an important feature which it had conjoined with the immunity from all fever particularly specified in the instances cited above, we conceive that the impression on unprejudiced minds must be adverse to the "perfect identity" of the cholera fever of England and the fever after cholera in Bengal. However, we have stated the recorded facts, and others may draw from them what inferences they please. We omit the two succeeding sections on the Appearances after Death, and on the Proximate Cause of the Disease. The usual cadaveric appearances and want of appearances are tolerably familiar—the proximate cause would not be a whit more so, did we dedicate the whole Journal to its consideration. It is singular that some persons have gravely affirmed that the epidemic cholera presents no distinctive symptoms during life, and is only to be certainly recognized by its cadaveric features. The absurdity of this dogma requires no serious refutation.

### Remote Cause of the Epidemic.

The Bengal Board observe that they have now arrived at a branch of their enquiry, in which new obstacles start up at every turn. The testimony of one contradicts that of another; facts themselves are irreconcileably opposite, that is they disprove any theory founded on a particular circumstance or set of circumstances; and finally the cautious reasoner is forced to take refuge in scepticism from the importunities of exclusive speculators. In this particular cholera really presents no anomaly, for the causes of former epidemics have proved as obscure as those of the prevailing one. The Board observe, and we think they observe truly, that their labours are rather calculated to prove our ignorance than to establish our knowledge; to prove in what the generation or dissemination of the disease did not consist, than in what it actually did. This is the modest, and, unfortunately, it is also the true state of the question.

---

left behind it on the banks of the Ganges, and carried that feature with it to the Thames and the Clyde. Would we find small-pox "invariably" confluent on one bank of a river, and "invariably" discrete on the other? No. But the fact is, that although some grand and invisible agents were at work to produce an epidemic disease, these agents called it forth as independently in each locality, as the grass grew and the water ran independently in each of the said localities. Neither the disease nor the causes of it migrated from one country to another. They were not *propagated*—they were *evolved*. Hence it was that the cause, whatever it may be, was modified in its effects by the locality where it operated. Why was the disease more prevalent or fatal in Musselburgh than in Edinburgh—in Ely than in Durham—in Paris than in London—in Newburn than in Haddington? Look at the localities, and see if they are similar. This we conceive is the philosophical view of the subject.

The term *propagation*, we maintain, is singularly improper and totally inapplicable to this epidemic and to its cause. *Evolution* is the fitter term, and ought to be substituted in all places for the absurd phrases of "marching," "travelling," and "spreading." Did cholera *march* from Newcastle to Haddington? If so, which was the road? From London to Paris? What *route* experienced its ravages? All we can safely say is, that the *evolution* of its cause took place in Haddington after it occurred in Newcastle—in Paris, subsequently to its evolution in London.

Vicissitudes of temperature and irregular seasons have been looked upon as the real causes of the epidemic. Troops were constantly exposed to the former yet they never received the cholera, until they manifestly came within the sphere of its influence. Whether the previously irregular seasons had any thing to do with its production, we cannot certainly tell; but it is certain that, being once set up, it has subsequently been evolved in defiance of season and in all temperatures, though season and temperature have both exerted a perceptible but inconstant influence over it. The blighted and noxious early crop of rice of 1817 has also been accused of originating the malady. Now the epidemic was in Nuddeea and Mymensing in May, and the seed of that calumniated rice-crop was only *sown* in June. So much for the damaged rice hypothesis. Of the origin then of the epidemic we know no more than this; that previously to its appearance the seasons were extremely irregular and unnatural, the people sickly to a great degree, and the country inundated. As the Board remark, the humidity of the atmosphere and the inundation of the Bengalese plains, assimilated the condition of the country to that of Egypt and the neighbouring parts of Ethiopia, lands stigmatised in every age as the original source and seminary of the plague. We may state broadly that all researches into the primary cause of the epidemic have failed, at least they have led to no more certain conclusions than those which we have mentioned. It remains for us to investigate the progress of its evolutions.

"Perhaps the most singular fact in the whole history of the disease, was the predilection which it shewed to spread in one particular direction.—From the remote period of its first appearance in the Eastern parts of Bengal, in the Autumn of 1817, to the hour of its arrival on the Malabar Coast, as has been seen in a preceding part of this report, its path was almost uniformly from East to West; and, if we may be allowed so to express ourselves, it seemed so bent upon pursuing this Westerly course, that rather than deviate from it in an opposite direction, it would for a while desert a tract of country, to which it afterwards returned under circumstances more congenial to its disposition.—Thus, although it appeared in the beginning of November, 1817, on both banks of the Jumna near Shergur, it did not then shoot Eastward across the Doab; but, leaving all on that side of the river untouched, spread far and wide through Bundlekund, and all the districts to the West.—In like manner, when it had reached Cawnpore in the following Spring, it shewed a marked aversion for Bareilly and the other tracts east of the Ganges; but readily stretched through the Doab to Agra, Coel, Delhi and Meerut; and thence far to the West, by Hissar, Jeypore, and the detached camp of the Rajpootana Force.—To give another instance.—Although Allahabad and the whole of that District, was largely afflicted in March, the infection was not thence communicated to Sooltanpore, Fyzabad, Oude, and the Districts bordering on the Gogra and Goomtee; but from the South East quarter, by the way of Tirhoot and Gorruckpore.—The case of the small Cantonment of Mulhye on the Eastern frontier of Tirhoot, which would seem to have received the disease from the West, and of one or two other places, in which the range of aberration was very limited, need not invalidate a rule deduced from an observance of a general course extending several thousand miles.

From knowing, that during the existence of former pestilences, the diffusion of the virus could be frequently traced to the motion of particular currents of air, it was natural to look for an explanation of this extraordinary regularity of progression in the prevailing course of the winds during that period.—Accordingly, upon reference to the various reports of the rise of the disorder in different parts of the Country, it was discovered, that in a vast majority of instances the wind was blowing from the East or South East quarter, at the time of its breaking out.—This may be stated to have been almost without exception the case in Bengal; throughout which the Epidemic arose in the Rainy Season, when the wind blows almost invariably from the South East.—In Calcutta, Nuddeea, and many other places, indeed, the influence of particular directions in the wind was so evident, as to have at length almost justified a prediction, that the abatements and aggravations of the disorder would certainly correspond with their alternations.—Thus in Calcutta, it declined in virulence and frequency as the Northerly wind set in, in November 1817; and again recurred with a South East wind in the following February.—Its re-appearance, again, in April 1818, was preceded by a continuance of wind from the North East: an uncommon quarter for that season of the year.—So in Nuddeea, the disease declined for a few days on the wind blowing steadily from the North; but no sooner had it again veered to the East, than it recommenced its ravages.—The same prevalence of Easterly and Southerly winds attended its progress through Tirhoot, Sarun, Behar, and Shahabad.—At Moozufferpore, Buxar, and Ghazeepore, this had been the prevailing wind for some time before its appearance.—In the camp of the Centre Division of the Army, the wind, which from the 21st of the preceding month had blown strongly from the West, suddenly changed round to the East quarter on the 7th November; and there are grounds for believing, that from that day the disease raged in camp.—With the Left Division again, the wind ranged from East to South, from the 1st to the 14th of April.—The Epidemic was with them on the 9th, and abated as the wind came round to the West.—The state of the winds is unfortunately omitted in the returns from the Nagpore Force.—With the Rajpootana Force they are stated to have been variable; but in Jeypore, Brigadier Arnold's Camp, Agra, and other stations of Central India, they were Easterly during the prevalence of the disease." 99.

To this rule there were exceptions, but they were neither numerous, nor of magnitude sufficient to upset the general law. The subsequent history of the disease has confirmed the fact of its tendency to appear successively in a direction from East to West. It is true that China and Japan have been visited, and no less true that, further to the East, it had only the deep blue sea to conquer, at least for many a league. Yet this Eastern irruption has constituted a sort of parenthesis in the history of cholera, and its progress has still been that which man and civilization have made before it, towards the Western Ocean and the Western Isles. From Asia our fathers came—from Asia came our arts, our learning, and our gods—and epidemic diseases, for example, influenzas, have generally begun in the East and terminated in the West, like the light of the sun.

Why should the cholera evince so marked a disposition as it has done to follow the course of rivers? Such was the query of the Bengal Board in 1820, and the answer that has been given is known to most of our readers. The rivers are the great channels of human intercourse in India, say the contagionists, and the course of rivers it accordingly pursues. The reply was specious—as a reply, almost unanswerable. Yet mark what has occurred in our own land. Here, rivers are not the great channels of human intercourse; there are others as great, if not greater, than they. Yet still, in England, the cholera has shewn its ancient preference for rivers, appearing on the banks of one after another, spurning all intercourse with intermediate spots. Nay it has raged on one side of a river, as in Southwark, and almost refused to pass a bridge which myriads of men were traversing.

" From the rise of the disorder on the banks of the Ganges and Burrumpooter to its arrival at the mouths of the Nerbudda and Taptee, it excited the surprise of the Medical observer.—Thus from Sonergong in the Dacca District, where the Epidemic broke out in July, 1817, it crept along the banks of the Megna to Naraingunj and Dacca; attaching itself chiefly to the ferries and market-places in its vicinity.—In like manner, it afterwards step by step advanced up the Burrumpooter; affecting during its transit the villages situated on both its margins. From the mouth of the Hooghly to its termination in the Ganges near Moorshedabad, the same peculiarity was observable.—The shipping at the New Anchorage, at Diamond Harbour, and along the whole channel as high as Hoogly was particularly affected; and almost every village adjacent to its banks buried many of its inhabitants.—In the Bhaugulpore district the propensity was so strong, that the virus scarcely ever spread into the interior, whilst it almost depopulated the low lands near the Ganges. Again, in the Autumn of 1817, Moozufferpore, and the villages along the Gunduk river in Tirhoot, and the station of Chupra on a branch of the Ganges in Sarun, were alone visited; while at a subsequent period the disease was thence communicated along the Gogra to numerous cities in the North East quarter of our territories.—From Allahabad upwards, along the channel of the twin branches there forming a junction, until the virus was lost under the hills, it wavered so little from the line of those rivers, that hardly a town or village lying remote from their course was brought within its influence. Without going further over our old ground, let us briefly state that the same rule held yet more unexceptionably in Rajpootana; through the province of Bundlekund; and all along the Nerbudda to the numerous branches of the Chumbul." 105.

Whether there be more occult causes than what we are about to mention for this preference, we cannot tell, but little doubt can now remain upon the minds of reasonable men of both their agency and efficiency. On the banks of rivers we always find the most populous, and in parts the most filthy communities, and on the banks of rivers we always experience in its intensity the agency of malaria. That malaria, or that undefinable condition of atmosphere which the neighbourhood of rivers presents, and the contiguity of rivers generates, has much to do with the local development of cholera, we have no more doubt than of the general truth of the Newtonian system. Of the nature of that influence we are as ignorant as of the nature of attraction, or the intimate essence of force.

The Board cite instance after instance, illustrative of the effects of low, damp, and unhealthy situations in aggravating the pestilence. It is true that even the healthiest places were occasionally invaded, but they were so less frequently and infinitely less severely than the others. We need not mention instances, but such was undoubtedly the fact. Not only were cities or villages in unwholesome situations most liable to suffer from the epidemic, but the most unwholesome places of those places bore its brunt. Has it not been so in England? The cholera is usually the tenant of lanes and alleys; like the fallen favourite, it is found in "the worst inn's worst room." Its causes and seats correspond most with those of ague.

"A striking instance of the good effects of airiness in affording immunity from attacks of the disease, occurred in the great Native Jail of Alypore.—Although this building contained several thousand persons, scarcely a case appeared in it; whilst the prisoners employed at the outposts, and labouring in the sun in cleansing drains during the day, and sleeping in mud buildings at night were very sickly.—So the Native Insane Hospital built in a low swampy spot, and such parts of the shipping as lay near the slimy banks of the river, were likewise peculiarly subject to the disease, whilst on the other hand, the dry Jail of the Court of Requests, containing nearly four hundred debtors, continued nearly exempt.

On proceeding upwards we shall observe the same unerring influence of situation.—In Nuddeea, high and dry places, and upper roomed houses, were more free than low and marshy spots, with luxuriant vegetation. In the Barracks of the European Regiment at Berhampore, of twenty-four casualties, seventeen took place in the four Companies inhabiting the lower range.—This range was very damp, and had in its vicinity to the North East an extensive swamp, from which an offensive stench proceeded.—The disease shewed no variety of appearance in Rajshahy, because the town of Nattore, and almost every village in the District, are equally surrounded by ditches, full of stagnant water, and filth of every kind. But in Malda, only the villages lying in the flats near the Mahanuddee River were attacked.—In Bhaugulpore, all the lofty open country escaped, whilst those parts most subject to fevers, suffered severely, as Calcapore, Rajmahl, Peealapore, Tarapore, and Luchmunpore.—The troops and followers in the cantonment of Carringur entirely escaped; although two villages situated immediately on their boundary ditch suffered dreadfully. But the cantonments were seventy-two feet above the villages. When the disease appeared first in Tirhoot, there had been a previous unusual inundation; and the low villages near the river were alone affected.—So in Sarun, the disorder arose shortly after a branch of the Ganges had rapidly fallen, and left the town exposed to the noxious smell proceeding from its large oozy bank.—The only place, accordingly, left unaffected was the Jail; which was clean, airy, and situated in an open space at a distance from other buildings. The same favorable localities nearly saved the Tirhoot Jail.—In the dry and well ventilated cantonments of Mullhye, only three instances of the disease occurred; while the neighbouring villages, which were remarkable for their filthy and close state, were so sickly, as to be deserted by the inhabitants. So in Poorneea, the lines of the Provincial Battalion and Jail were quite exempt, when the disease was extensively fatal in the town.—In the valley of Napaul, all the villages enjoying a pure air on the slopes of the hills remained free; whilst the towns of Khatmandoo, Patun, and Bhatgoon, in the vicinity of which there was much putrid matter, and many channels of stagnant water, were severely visited.—In Benares the disease was chiefly confined to the lower classes of Natives living in wretched huts.—In like manner, at Lucknow, the cantonments, built on a dry and sandy soil, elevated somewhat above the general surface of the country, enjoyed nearly entire immunity; when the low and crowded city was greatly ravaged.—That nothing in the condition of the Sepoys themselves, was the cause of this good fortune, was very evident, both here and at Mullhye, by their becoming affected upon proceeding on guard to places within the range of the unhealthy action." 115.

We remember hearing Dr. Roderick Macleod remark in the Westminster Medical Society, that the jails had generally been the last places attacked in the town where the cholera prevailed. The Doctor is a stanch contagionist, and he accounted for the fact, or asserted fact, by supposing that the communication between the prisoners and town's-people being restricted, the disease was consequently slow in making its way into their aristocratic ranks. Where Dr. Macleod found the facts in question we do not know, and therefore we must take them as we get them; but we may venture to observe that the preceding quotation from the Report before us, supports neither them nor the inferences founded on them. This shews the danger, we had almost said absurdity, of forming or trusting to partial views.

Many instances of the apparent effects of locality are related by the

Board. We cannot find space for them, but we may state broadly that they fully prove the general law, that damp situations and crowded places were particularly obnoxious to the epidemic. Still it must not be supposed that these suffered exclusively. As a general rule, there can be no doubt that marshy spots are peculiarly prone to give rise to intermittents. Yet who does not know that ague and remittent fever are sometimes endemic, where the keenest investigation can detect no apparent cause? If this be the case with an endemic malady, how much more likely is it to be so with one which blends the characters of an endemic, epidemic, and in, perhaps, some degree contagious disease, together!

"Amidst so many proofs of peculiarities of situation influencing the epidemic, it is not intended to deny, that in the vast range which it took, there were many places, in which it not only failed in observing this rule, but even seemed to go right in the face of it. Thus, there were even whole districts, as Cawnpore and Juanpore, in which it affected all parts alike, without reference to their being high or low, damp or dry:—Others, again, in which, though it affected particular lines and villages, its liking for those parts was not to be explained on the supposition of greater general insalubrity, than that of the spots avoided by it. Lastly, there were even instances of its shewing a preference for dry and wholesome, over damp, and aguish situations: as in Allahabad, in which the lines of the Native Artillery Details, lying in the low and swampy suburbs of the city, and much exposed to putrid animal and vegetable exhalations, were alone spared; while those of the European Invalids, which stood high and dry, and all around, was largely infected with the virus. But these exceptions were comparatively few in number; and should not be allowed to affect the general conclusion, founded on the large body of affirmative evidence previously adduced; that the power and range of the epidemic, the poison being once afloat, were always increased by such causes, as experience has shewn to exercise a marked agency in the generation and dissemination of Jail, Bilious, Remittent, and Intermittent fevers!

Is the entire immunity of lofty and mountainous situations attributable merely to their being, from their greater altitude, less surrounded by those vapours, and less exposed to those extrinsic circumstances, which subjected the lower lands to the influence of the disease? Or, was the peculiar poison of the epidemic confined to the inferior strata of the atmosphere? That this was not invariably the case, was proved, by the pestilence getting over the high chain, which divides Nypaul from Tirhoot, and Munnipore from Sylhet.—But the difficulty, with which it appeared to ascend these heights, and the perfect immunity of the elevated fortresses of Rhotass, Adjeegur, and Kallinjur; of Kumaoon, the Dhoon, and the hilly parts of Rajpootana, while all around them was infected, would rather incline us to believe, that there was something in the air of elevated situations intrinsically hostile to the existence of the poison." 122.

### Of the Contagious Nature of the Disease.

Such is the title of the sixth section, an interesting and an important one. Let us see what was the impression on the minds of the public and of the profession, in the districts where the disease first manifested itself with a virulence which it has never since displayed. There were then no theories to be supported, no interested motives, no party spirit. Men might have been wrong in forming their opinions, but their errors would be merely those of judgment, not tugged in one way or another by the influence of the feelings or by factious, not to say sordid motives. The Board put a bold face on the matter at starting.

"If, by contagion, is meant the communication of the disorder from person to person, by means of contact, or close conversation; then, in this strict sense of the word, Cholera is certainly not contagious.

In the absence of all positive proof, such a conclusion might have been fairly drawn, from its being observed, that in no quarter of India, during the time in which it was so sadly scourged by the disorder, did its infectious nature form any part of the popular belief.—Amongst a rude and superstitious people, the unexampled mortality caused by it, was, according to the fancy of the individual, ascribed to fatality, to the agency of malignant spirits, or to the anger of an offended Deity: but it does not appear to have been once suspected, that its amount was increased, or diminished, by the free, or restrained intercourse of men. It may be said, in diminution of the weight here attached to the popular persuasion, that the opinion of the vulgar is usually founded on misconception, or guided by caprice; and is therefore of little, or no value.—This is no doubt true, in respect of subjects, either foreign to their interests, or too recondite for their understandings.—But, in matters of daily observation, and especially in those narrowly concerning the interests and safety of all, there is perhaps no fairer criterion of truth, than the common judgments of mankind.—The progress of any generally fatal disorder is exactly of this description; and accordingly we find, by looking into the histories of all the great epidemical and infectious distempers, to which the human race is subject: as the plague, small-pox, measles, and scarlet fever; that the people were never slow to discover their true nature; and ordinarily passed such judgments regarding them, as corresponded, not merely with the opinions of more learned observers, but with the truth itself.—So it is in the case of the present epidemic. The whole body of the Medical Officers in Bengal, who have had an opportunity of seeing, and remarking on the disease, without a dissenting voice, concur in declaring, that it is not contagious."* 124.

Popular opinion, however, is fallible, and the unanimous opinion of medical men should not be deemed conclusive, unless supported by facts which themselves bring conviction to a reasoning mind. Let us see then how facts bear in this particular instance. The disease could not possibly have originated in contagion, that is obvious. If evidence is to be made the foundation of induction, and when we discard that we take refuge in fancy, the disease commenced simultaneously, or nearly so, in several places. Now it is conceivable that a disease may spring up in a particular spot, and be subsequently propagated by means independent of those which produced it. But if the malady appears not in one, but in several places, it is then more consistent with the principles of ratiocination and the dictates of common sense, to imagine that the causes which gave it birth, are probably operative still in its evolution. Cholera differs from diseases which we know to be contagious in the remarkable tendency to wear itself out in a definite, and that a comparatively short time. We may select some examples of this general law.

"But, again, the whole habitudes of the disease, when once it had entered a town or camp, proved, that it was not kept up by infection. Instead of daily increasing, and being perpetuated by the very means on which it fed; it invariably ran a regular course of increase, maturity, decay, and extinction.—Thus, in the Centre Division of the Army, it began on the 7th of the month; was at

* "To this unanimity of conviction there was originally one exception; but, from more extended experience that individual has since modified his opinion."

its height from the 16th to the 22d; declined to the end of the month; and finally disappeared about the 2d or 3d of December. So in the Left Division of the Army, it commenced on the 10th of April; was at its full in the middle of the month; declined from the 21st; and died away before the beginning of May.—The case of the Nagpore Force is somewhat different.—The corps composing it fell at once into a medium already fully impregnated with the poison; and, without passing through the primary stage, or that of increase, immediately had the disorder in its most violent form.—The only effect of this change of circumstances, however, was to diminish the usual period of the revolution; for the disorder which began on the 31st of May, had abated previously to the 5th, and nearly disappeared soon after the 18th of June. In the Rajpootana Force, the sickly period was still shorter.—The disease appeared on the 14th of September; and continued violent to the 20th; after which it gradually declined till the 1st of October, when it wholly disappeared. Lastly, in the Hansi Division, it observed the same regularity of course; beginning on the 6th of August, increasing in severity for a time, and gradually becoming extinct towards the end of the month." 127.

In answer to this it has been urged, that if cholera or any other pestilence were to proceed till no more subjects were left for it, the world would be depopulated. The objection is specious but unsound. It has been wisely ordained by the omnipotent and omniscient Creator, that all men should not be liable to all maladies—that one should be insusceptible of small-pox, another of scarlet fever, another of plague, and so on. If this were not so, undoubtedly the world would be soon depopulated. But still a large portion of mankind do seem to be susceptible of those diseases, which the common experience of common men has decided to be infectious or contagious, or both. Setting aside the effects of vaccination, we find this the case with regard to small-pox, measles, scarlet fever, nay even to the plague. The tendency of these maladies is to attack, and so far as their virulence extends, to destroy the many individuals who can suffer from their poison. But accident or design, (and we are of that sect who think accident acting beneficially and often, is probably design) have interposed barriers to the extension of these evils, and checked them in their pernicious career. Thus vaccination counteracts the diffusion of small-pox, diminution of temperature that of plague. Take away these counteracting agents, and we do not observe in the diseases thus abandoned to themselves a tendency to run through a brief career, exhibiting in the limited space of a fortnight, or a month, a manifest series of changes, a period of increase, an acmé, and a rapid decline. Plague exhibits such stages it is true, but plague appears in a certain temperature, and by a very low or very high temperature it is extinguished. It is certain that this law of cholera is not absolutely conclusive against its contagiousness; but, inasmuch as it constitutes a most essential difference between cholera and diseases manifestly contagious, it serves to destroy the analogy between them, and makes us hesitate in imagining that their propagation can be governed by the same laws.

In contagious diseases mankind seem to have hit upon some sort of quarantine by common and universal consent. In the present instance, no such consent has been observed, and in India quarantine was never deemed necessary. Again, in contagious diseases quarantine has on the whole displayed some powers in preventing the importation of the disease against which it was directed. Against cholera, we need hardly say that it has proved entirely impotent and futile. Here again is a striking difference between cholera and diseases avowedly contagious.

In contagious diseases, the great bulk of the medical men who witnessed them, were forced to bow, whatever might be their inclination, to the majesty of truth; they were compelled, in short, to acknowledge the force of facts in favour of their contagiousness. How different the case has been with cholera. Where the disease was not, the contagionists have usually out-numbered their opponents, but so soon as an opportunity of personal observation and experience was afforded, round went the wind, and the anti-contagionists became the overwhelming majority. This has been the case in Europe, and very remarkably so in this country. In India we find the same thing take place, with this difference only, that as few contagionists ever existed, so very few could possibly be converted. Let us now look at some of the circumstances which produced so deep and so universal a conviction of the non-contagiousness of the epidemic on the part of our Indian brethren.

"The opinion of the medical observer was, in all situations, founded upon his noticing the following facts.—In his attendance upon patients labouring under the disease, he did not find himself, or his assistants, more liable to be attacked by it, than such persons, as had no communication with the infected.*—He could not attribute his escape to the effect of precaution, for he took none; nor to the limited nature of his intercourse with his patients; for the disorder, and the remedies employed in it, were such, that he was obliged to be constantly handling the body of the sufferer, and could not with safety leave his bedside, during the height of the attack.—It might even be said, that in every case the patient and he breathed upon one another: so that, had the effluvium exhaled from the lungs or skin of the patient, been truly infectious, even in the slightest degree, he could have had no chance of escaping.—Next he saw, that where one member of a family was ill, the others were not more liable to get the disease, than an equal number of individuals picked out from the general body of the community.—This must be taken with some allowance.—Sometimes two or more members of one family were seized; but in such cases, they were generally all taken ill together; were living in the same unwholesome situation; and had been previously exposed to some manifestly strong exciting cause: as the eating of noxious food; sudden vicissitudes of temperature; and the like.—In the rare instances, in which one fell ill at some distance of time after another, if we do not chuse to consider the concurrence as purely accidental, we shall be at no difficulty to explain it, upon remembering the depressing influence of fatigue, fear, sympathy, and grief—all powerful predisposing causes.†

---

* "This is a very striking fact. From a medical list, consisting of between two hundred and fifty and three hundred individuals, most of whom saw the disease largely, only three persons were attacked, and one death only occurred.—The fatal case took place at Barrackpore, a station very little visited by the epidemic; the two others, which were not severe, occurred in the Centre Division of the Army. There too, one of the Surgeons of His Majesty's Corps was cut off; the only Medical Officer belonging to the King's Service known to have been affected.—In Nagpore the Medical Staff remained for several days, night and day in the hospitals, and yet all escaped."

† "A very striking example of the noncommunicability of the disease by contact was afforded in Colonel Gardner's Irregular Horse, which was attacked at Khassgunj in the Doab in August 1818. No two men were seized in the same

In camps, where the general body was more compact, and the sick more numerous, and crowded in smaller space, there was still ampler opportunity of confirming the truth of these observations. In no one instance were the dooley bearers, native compounders, or any other part of the large hospital establishments then necessarily kept up,—although all were often so hard worked as to be scarcely able to stand from fatigue—more sickly than other descriptions of followers; nor did the soldiers, who constantly flocked to the hospitals, to see and watch over their sick comrades, appear by that means, to be more susceptible than others, of the disease.—Nor were those patients, who were ill of other disorders, although always surrounded by persons in every stage of Cholera, therefore more liable to be attacked; unless perhaps an exception be made in favor of convalescents: a class of persons always, from debility, much predisposed to fall into fresh disease. In the Centre Division of the Army all this was particularly remarked; and during the week in which the Epidemic raged with so much fury; when the camp was a sick ward, and every tent was filled, or surrounded with the dead and dying; the Officers suffered comparatively very little. From a number that could hardly have fallen short of three hundred, only five or six deaths occurred. And it should be remembered, that at this time Officers of all descriptions were equally exposed with the Medical men; for the sick had become so numerous, that even the services of all were insufficient to tend them with proper care, and duly administer the requisite remedies." 132.

When the epidemic has attacked one member of a family, it appears to us that it has generally evinced a disposition to attack one or more of the rest. To this rule there have been numerous and striking exceptions; such, indeed, as have induced observant men to assert, that no such disposition exists. But, granting the fact to be as we have stated it, we cannot regard it as furnishing any thing like conclusive evidence in favour of contagion, and for these reasons:—First, when one member of a family has been attacked, the others have usually suffered in such rapid succession, as to lead us to suspect some general cause acting upon the whole; secondly, supposing a disease to be both epidemic and endemic, which cholera unquestionably is, all the individuals of a family are exposed to the same general and partial influences which acted on the one, and, therefore, are more likely, on these accounts, to be attacked than other individuals or other families; thirdly, all use the same food, and are nearly similarly circumstanced in regard to clothing, lodging, habit of body, and predisposing causes; fourthly, fear, alarm, depression, are perhaps the most powerful of all predisponents or excitants of cholera, and to these great agencies families are particularly open, when one has already been cut off before their eyes. These considerations should make men hesitate, before they jump to the conclusion that contagion has been proved, because several members of a family have suffered from the epidemic. When common catarrh is prevalent, that is, epidemic, practitioners, nay, the vulgar, know that one in a family is seldom attacked

---

hut, although from twenty to thirty troopers slept in each. A case exactly the opposite of this occurred in Lord Hastings' camp at Gorruckpore. A Sepoy died of the Pestilence. Five of the Corps, who had shewn no signs of illness, were employed to cary the body to the grave. They were all seized with the disorder during the ensuing night; and all died. This no doubt looks very suspicious; but then we know nothing of the concomitant circumstances; which as in other instances of apparently dubious origin, might have been sufficient to do away every suspicion of the agency of Contagion."

alone. So it is with every disorder in which epidemic or endemic agencies exert an influence; indeed it has been less the case with cholera than might reasonably have been anticipated. Where several in a family have been attacked in England, they have almost universally been crowded and destitute; whilst, on the other hand, the instances, and numerous they are, to the contrary, have been in places where cleanliness and ventilation were more attended to, and such abject poverty has not existed. For the same reasons, we imagine, this extension of the disease was less remarked in the military hospitals and in camp, than amongst the crowded and miserable villages and filthier suburbs of towns. In England, also, the epidemic has attacked several members of a family in the lanes and alleys of London, Sunderland, and Newcastle. But wherever persons in easy circumstances have been the victims, it has *not* spread among their friends and relatives. This was the case in the neighbourhood of Haddington, where one or two servants were affected—at Hillhead, near Kirkintilloch—in London, in the instance of Lady Wyndham, Lord Holland's servant, and on other occasions. These remarks are, we think, a sufficient answer to those, who rest their proof of contagion on the circumstance of several in a family being frequently infected. It is on such proofs as these that the Board of Health of Ely have founded their declaration, that cholera is in some degree contagious. That this is the case, under certain circumstances, we ourselves believe, and have always maintained; but we think that it is neither fair, nor safe, nor philosophical, to bottom that belief, still less to found any public declaration on this particular fact. Nothing is so dangerous as forming a sweeping opinion on one circumstance. To do this, is to abandon the advantages derived from our powers of reasoning and comparison. The Bengal Board relate several instances of striking immunity of particular bodies of men, in the midst of the disorder, or having the disorder in the midst of them.

"From the centre Division a few days previously to the breaking out of the Epidemic, a small force, consisting of 4 troops of the 7th Regiment Native Cavalry, 3 Light Companies of Sepoys, and the Dromedary Corps, was detached on particular service in the neighbourhood. A short time afterwards, the remaining squadron of the Corps of Cavalry was sent as a reinforcement from the great camp, in which the disease had then got head. It carried the virus along with it; and actually lost several men, after its junction with the foregoing detachment; which nevertheless remained perfectly healthy throughout.

But, there is yet a still stronger instance of the possibility of a diseased body joining a healthy one, without thereby communicating the infection to it. On the morning of the 11th of May, 1818, a detachment of 90 men of the 1st Battalion 26th Native Infantry marched from an inferior post, to join the main body of troops then encamped at Saugor. After an ordinary march, it halted in perfect health, half way; under shelter of a few trees on the banks of a small lake; situated in the midst of an open space, about three miles in circuit, and surrounded by low, woody hills. The whole remained well until the fall of night; when Cholera broke out amongst them. The first man was taken ill at midnight, and died in half an hour. Several others fell sick within the next few hours; and before sun-rise, twenty out of the ninety were overtaken by the disease. Although the Saugor camp was distant only five or six miles, the detachment was too weak to move without assistance. The sick of the Sepoys and followers were therefore carried in, in carts and doolies sent from the main body; but before 11, A.M. when they got to their ground, five were already dead, and two others moribund. Next morning, a man of the same party was seized in the act of scouring his accoutrements; immediately became insensible; and expired in a few minutes. During the three succeeding days, several others were taken ill; and before the end of the week, of the whole Detachment, there was not a single man, but was sent to the hospital, labouring under Cholera, or other modifications of bowel-complaints. The men of this party mixed promiscuously with those of the Saugor Troops; and yet of the latter not one individual got the disease.

An instance of the same kind occurred in the Hansi Division; except that here the party, which escaped, went into the infected medium, instead of the pestilence being carried amongst them. When the disease was at the worst with the troops composing this Force, Casement's corps of Irregular Horse entered the camp, and continued with the Division during the remainder of the service: yet it did not at all suffer.

It may be supposed, that in these cases, the persons who escaped, owed their immunity to their not having been long enough exposed to the poisonous matter; or to some incidental peculiarity in their condition, for the time being. But, then, we shall find, that the same irregularity obtained, where those remaining unaffected were for a long period surrounded by the supposed infectious atmosphere; and in all respects similarly situated with others, who suffered severely. Of the latter, a remarkable example was afforded in the Left Division of the Army, whilst under the influence of the disease. Here the 7th Regiment of Cavalry, and the 2d Battalion 13th Regiment of Native Infantry remained entirely exempt;* and the 2nd Battalion 1st Regiment had only three mild cases: while the 1st Battalion 14th, and the 2nd Battalion 28th Regiments were greatly affected. The same partiality of affection here took place among different classes and descriptions of troops.—The Goolundaz, gun lascars, and miners were mildly affected; while the pioneers, drivers, &c. who had undergone the same vicissitudes of weather, and fatigue, were not at all touched.—Some corps lost more than a hundred, others only three or four men. This could not arise from separation, or difference of situation and diet; for all used the same food; and there was constant intercourse, and daily change of ground.—The same observation may be extended to the Rajpootana Force, of which the right suffered more than the left portion.—In Furruckabad Lines, the Jail and the Artillery Barracks, the former containing six or seven hundred prisoners, subject to great privations, and daily worked in the sun upon the roads, and the latter inhabited by 100 Europeans and 250 Natives of the 12-pounder Experimental Brigade, had not a single case; whilst the Levy Corps suffered severely. The case of the Native Artillery Lines at Allahabad, which escaped, although in the very centre of the pestilence, has been already alluded to.—But at this station, there was something yet more to our purpose. For of 400 Supernumerary Invalids, assembled there for examination by the annual Invaliding Committee, not a man was affected, although they were living, under perfect similarity of circumstances, in the Lines of the Regular Invalid Battalion, the men of which, in fourteen days, had 50, of 680 their total number, sick of the disease.—So, while the disease raged virulently at Banda, not a man belonging to the 2nd Battalion 3d Native Infantry stationed there was affected.—At Hutta, again, a healthy town on the banks of the Sonar in Bundlekund, the Epidemic committed such ravages, that the inhabitants fled, and took refuge in the neighbouring villages; and so virulent was the poison, that three Sepoys and seven camp followers of the 2nd Battalion 1st Regiment were seized, merely on that Corps

* "It is true, this and other instances to be cited, may be in some measure accounted for, by the Corps having earned exemption by previous exposure to the influence of the disease, whilst forming part of Colonel Philpot's Detachment from the Centre Division."

marching through the place.—And yet, the disease never appeared amongst a Company of Sepoys, or their followers, then in the fort, which was divided from the town only by a broad street.—What here served to skreen these men from infection? Certainly no suspension of intercourse between the town and fort; for this always remained free; much less superior salubrity of situation, for the fort was small, and crowded with buildings, and the town high and open.—Thus too, whilst the disease raged in Saugor, and in the lines of the 1st Battalion 26th Native Infantry about a mile and a half distant; not a case occurred in the fort in the centre of the town, which was then garrisoned by 200 men of the 2d Battalion 1st Regiment.—In like manner, in Kotah, three Companies stationed in the fort escaped entirely; whilst one hundred persons were daily perishing in the town.—And at Muhedpore, when the Epidemic prevailed in the vicinity, and was daily attacking a detachment of Bengal troops, consisting of part of the 1st Battalion 6th Regiment Native Infantry, two *Rissalus* of Skinner's Horse, and 1500 Camp followers, it entirely spared a body of 500 of Holkar's Reformed Horse; although the two camps closely adjoined, and a man, who had been sent in from the Bengal Division after getting the disease, went through every period of it amongst the healthy Mahrattas. If the virus were capable of being procreated by Contagion, surely the poisonous particles emanating from the body of this infected person, would have been sufficient to support and diffuse it all around.—For, this body, having an infected person in the midst of it, was, except in respect of numbers, then in the precise situation of the larger Divisions of the Army; in which the disease always began with one or two unconnected cases; slowly and gradually creeping on to inveteracy.—With it, all that was wanting, was the peculiar disposition to take on, and keep up the diseased action; and this being absent, the supply of vitiated matter, which, in small-pox and other distempers, confessedly contagious, would have sufficed for its continuance, proved quite innoxious.

We should fall into endless repetition, if we went on citing the great variety of evidence, which might be drawn in support of the doctrine of non-contagion, from the general progress of the disease, through the districts and towns successively visited by it.* Let the reader only call to mind the innumerable detached spots, which remained free, when all around was sickly; and remember, that in no single case, was any restraint placed, on free intercourse between the healthy and diseased; and he must come to the conclusion, that the Epidemic was totally independent of the common laws of Contagion." 139.

As we do not wish to advocate one side of this question merely, we shall mention the facts, supposed to be favourable to the idea of contagion, which are mentioned by the Board.

" But as it cannot be denied, that there were some circumstances in the manner in which the disease arose both with the Hansi and Centre Divisions, militating against this opinion, let us, before leaving this part of the subject, examine them a little more narrowly.

The persuasion of all the Medical Staff present with the Hansi Force, regarding the mode in which it received the infection, has been already quoted. It consisted in the belief, that it was communicated by the Meerut Detachment, who got it on passing through Delhi, at the time of their crossing the Jumna.—

---

* " One case is too remarkable to be passed over. Of the cluster of islands lying near the main land, as the Ganges discharges itself into the Bay of Bengal, Sundeep, a large and populous place, remained quite free; whilst those of Deccan Shahbaspore, Huttiah, and Bomney were ravaged. There was nevertheless constant and great intercourse between all." 138.

The opinion of so many intelligent persons, is no doubt entitled to much respect; and would entirely set the question of Contagion, in that sense of the word in which by infection is meant the communication of the disease from one large body to another large body, at rest; were it not counterbalanced by some circumstances of an opposite tendency.—If we believe, as stated by one gentleman, that before the junction of this detachment, the disease had already found its way to several places intermediate between Delhi and Hansi, and Delhi and Kurnaul, we can be at no loss to account for its rise in Camp, without resorting to a belief in Contagion.—For, the Camp presented to the Epidemic exactly that face of things, which we know to have been always particularly affected by it; namely, a large body of men collected within a narrow space.—We are accordingly told, that previously to the junction of the Meerut Detachment, one or two cases had actually occurred amongst the Camp followers.—Admitting this to have been the case, the certain consequence of the great additional stock of pestilential matter imported from Delhi, would be to aggravate, and widely diffuse the disorder; and the whole mischief might very naturally be placed to the account of the new comers, by persons unaware of its having begun to operate, though in a less degree, previously to their arrival.—However this might have been, there were no grounds for supposing, that, even here, the disease was communicable from person to person; and the medical Officers are unanimously of opinion, that it was certainly not so.

The case of the Central Division is encompassed with still greater difficulties. The main body of this Division crossed the Jumna at Shergurh on the 28th October; and after one or two days halt, marched in a North Westerly direction, towards Loharee, Nuddeeka-gaon, and Terayt.—A detachment, composed of 5 Companies of the 2nd Battalion 13th Regiment Native Infantry, and two Companies of Pioneers, was left behind, in charge of the bridge of boats thrown over the Jumna.—It was here, that the Epidemic first shewed itself. A few cases appeared as early as the 2nd of November, in some troops then passing over; but on the 5th, the disease became common in the Detachment on guard.—On the 9th, this Detachment joined the main body of the Army at Terayt; and it is declared by some of the Medical Officers then on the spot, that during the two immediately subsequent days, the disorder was first observed in Camp.—In support of the opinion, that this Detachment brought the disease into the previously healthy Division, it is added, that the 2d Battalion of the 13th Regiment was brigaded to the left of the 1st Battalion 24th Regiment Native Infantry, and the 1st Battalion of the 24th to the left of the 2nd Battalion 11th Native Infantry; and that the 24th was attacked before the 11th. Lastly, in further proof of the communicativeness of the virus, it is affirmed, that the previously healthy villages, and among other places the town of Sumpter, got the infection from the Division.—This is the amount of facts in favor of the disease being capable of transmission by a large body from an infected, to a distant salubrious atmosphere; and thus communicable by contaminated, to healthy individuals.

It must be confessed, that this evidence is very strong, and would prove quite conclusive of the matter, were it to remain unshaken. But, unfortunately for the hypothesis meant to be established by it, there is hardly one item of it, that is not opposed by circumstances of a contrary tendency.—And, first with regard to the appearance of the disease in the main body of the Division, the testimony of different individuals, is so much at variance, as to be quite irreconcileable. Of twelve Medical Officers, who have given replies to the queries on this point, one states the disorder to have broken out on the 6th; two on the 7th; one on the 8th; two on the 9th; one on the 10th; four on the 11th; and one on the 12th. This discrepancy, however great, is easily explicable, when the insidious nature of the disease at its first onset is taken into account; and when it is recollected, that the sphere of each individual's observation, would hardly go beyond the Battalion, immediately under his charge.—But, how are we to reconcile the assertion of its having appeared on the 6th, 7th, 8th, or even the 9th, with the assumed hypothesis of infection from the Shergurt Detachment.

In like manner, the fact of the Division communicating the virus to the villages lying on its route from Terayt to the Betwah, is equally disputed. It is held by some, that those villages were not at all affected; by others, that they had the disease independently of any intercourse with the Division; and by a third party, that the latter invariably carried the infection along with it.—One gentleman, having several times sent his Native assistants into the district, was uniformly told, that the pestilence was not amongst them.* Another declares, that all the neighbourhood was sickly: the dry and clean town of Sumpter, equally with the low and filthy village of Nuddeegaon. A third, that the Army having on the 13th marched from Terayt to Talgong, the inhabitants of the latter place got the disease the following morning; and a fourth, that Sileia was first affected, on being entered by the Division on the 19th." 143.

In respect to the Meerut detachment communicating the disease to the Hansi force, it is quite ridiculous, when it is admitted that the camp of the latter "presented to the epidemic exactly that face of things which we know to have been always particularly affected by it—*a large body of men collected within a narrow space.*" Add to this, that some cases had occurred *before* the Meerut division joined the camp of Hansi. Every person of enlightened views will see that the congregation of troops did not bring "additional stock of pestilential matter" from Delhi; but produced additional pabulum for the epidemic or endemic cause to work upon. As to the centre division, the accounts contradict each other; and if they were all accordant, it amounts only to the exploded and fallacious doctrine of *post hoc ergo propter hoc.* Two days after a detachment joins an army, the disease appears in that army! How often has this kind of argument been rendered futile in Europe during the present epidemic!

It has been observed in this country that persons coming to visit their relatives or friends dead or dying from the malady, have in several instances been themselves cut off. To a superficial examiner this might appear strong evidence in favour of contagion. But then it must be remembered, that these persons expose themselves to the epidemic and endemic influence, whatever it may be; and, in the latter instance, have superadded to this the predisposing causes of fear, fatigue, dejection. If a person, after visiting a place where cholera is, were not only to have the disease himself, but if, on his return, his fellow townsmen also were infected, then indeed all must own that a case in favour of contagion was made out. But how seldom has this been done? under our own eyes, never. Many assertions of this kind have been made, but when properly sifted, they have proved mere dust, fitter to blind than to clear our view.

"Under such extreme contrariety of testimony, it would be vain to attempt drawing any certain conclusion.—This much, however, may be affirmed, from a review of the whole progress of the epidemic in this quarter, that the infectious medium, in whatever it consisted, was confined within a very circumscribed circle;

* "Col. Philpot's official reports state positively, that the thinly scattered villages on the route of his march remained unaffected; while his detachment was suffering very severely."

and was very slowly extended to healthy parts of the atmosphere.—If, setting aside the circumstances militating against it, we take it for granted, that the infection was truly received by the Centre and Hansi Divisions from the Detachments above-mentioned, we must believe, that the disorder, although not communicable by contact from person to person, was so from one large body to another large body; and that, wherever the poison got head amongst a number of men, it assumed some new quality, so as, when mixed with the atmosphere, to become infectious.—What constituted this additional quality, we cannot pretend to determine; but, in support of its existence, we may quote the predilection of the epidemic for cities and camps; the infection of the Left Division and the Nagpore, and Meerut Troops, immediately after entering into the diseased medium at Jubbulpore, Nagpore, and Delhi, and the similar case of the troops and followers in attendance upon the Governor General, being attacked shortly after communicating with an infected village, in the Gorruckpore district.—To the same account may be placed, the progressive march of the disorder from one part of an infected place to another; as in the Centre and Hansi Divisions; and more particularly the Rajpootana Force, in which the virus seemed to be regularly propagated from Corps to Corps.* In some instances, the suffering body would appear to have sickened immediately upon coming into the poisonous medium; as was the case with the Nagpore Troops, who were affected on the very day in which they encamped at the infected village of Gaongong.—But, more frequently one or two days would seem to have been requisite to bring the virus into action. Thus the Meerut Detachment entered Delhi on the 29th, and was not affected till the 31st; thus too, the Hansi Troops had not the disease till the 6th, the day after the junction of that Detachment.—Again, by those abetting the opinion of the disorder being communicated to the Centre Division by the Shergur Detachment, it is stated that the first cases occurred on the 11th, two days after its junction. Lastly, the followers of the troops in personal attendance upon the Governor General in April, first suffered on the 23d, three days after encamping near an infected village." 146.

This closes the section on contagion, and with it is almost terminated this article. With regard to contagion, we may say, as the Board say on another occasion, that the proofs in favour of it in fact amount to nothing, certainly to nothing satisfactory or decisive. That they frequently are calculated to breed suspicion, we freely allow, and they check the precipitancy of those who unhesitatingly declare that cholera is under no circumstances contagious. That contagion can be generated under certain conditions, we firmly believe; but what these are, we do not precisely know. That filth and crowding can call it into life, is probable enough, but whether other circum-

* "The line of this Force faced nearly North and by East. The troops were arranged in the following order, commencing from the left. The 1st Battalion 28th Native Infantry: 6th and 7th Companies Pioneers; Goolundaz and Gun Lascars; the Park in the Centre; 5 Companies 1st Battalion European Artillery; 1st Battalion 27th Native Infantry; Squadron 2d Regiment Cavalry; 2d Local Cavalry; in the rear of the Park were 415 Ordnance Drivers, with lines of Officers' tents intervening. The 1st Battalion 28th and Goolundaz were attacked on the 14th September; on the 15th a few cases occurred amongst the Pioneers and Lascars; but among them and the Gunner Drivers they were not numerous till the 19th. On the 18th the Squadron of Cavalry was attacked; and on the 20th, the Local Cavalry, and 1st Battalion 27th N. I. It must, however, be remembered, that the ground was drier on the right, than on the left of the line."

stances and what, can do the same, we feel ourselves unable to determine. Perhaps at some future period, when party spirit has declined, when more facts are before the world, and a calmer and more impartial consideration of them is adopted, the obscurity that veils this subject may be dispelled, or at all events diminished. Till then we adhere to contingent contagion, the safest, most philosophic tenet, as squaring with most facts, and now the most prevailing opinion.

Passing over the section on the predisposing and exciting causes, which contains nothing remarkable, we shall linger for a moment at that which tells of the mortality caused by the disease in Bengal. It appears that the accounts of the mortality in the towns and villages are not to be relied on. In addition to those sources of delusion which European communities have displayed, there were many of native growth, which we need not stop to mention. We shall confine the few extracts we intend to make, to those which give us information which deserves to be depended on. It appears that, in Bengal, males were liable in a greater proportion than females, whilst children and infants almost escaped. In this country, women have suffered more than men, and children in a greater proportion than in India. In Paris it has been different. After displaying the reputed and computed mortality in different cities and districts of Bengal, a mortality that makes a very formidable appearance, being formed of liberal allowances of sums of 10,000, 8,000, and so on, the Board state the ascertained loss in the army. Even here it is probable than inaccuracies have occurred, but they are not to be looked on as invalidating the returns.

"Let us next examine the amount of the loss sustained in the different Corps of the Army.—In the Centre Division the mortality was, on conjecture, variously calculated at five, eight, and twelve thousand.—The truth is, that in all cases, it is next to impossible to compute correctly the exact amount of the vast variety of persons, constituting the class of Camp followers with an Indian Army; and amid the confusion and desertion occurring in the Centre Division during the sickly period, even had its original strength in numbers been known, the real extent of its loss could hardly have been ascertained. The mortality was undoubtedly greatest from the 14th to the 19th of November; and it was calculated, that, at the very least, five thousand of all classes perished during these five days. The armed force consisted of 3,500 Europeans, and 8,000 Natives. Of the former, 230, and of the latter 534, died. The average loss of each Battalion was computed at 50 men; but this is mere conjecture, as no accurate returns were obtained. In some Corps the number attacked was very great. Thus the 1st Battalion 8th Native Infantry had 350 seizures, and 40 deaths; and the 3d Regiment Cavalry 154, and 18 deaths. In the 2d Battalion 1st Regiment Native Infantry, 48 Sepoys, and 25 followers died, between the 10th and 26th. From the 2d Battalion 11th Regiment Native Infantry, 268 cases were admitted, of whom 43 died.—In the Rocket Troop, 27 Europeans, of whom one only died; and 87 Natives, of whom 18 died. Of 2,000 of all classes, attached to the King's 67th Regiment of Foot, 90 died. The mortality amongst a given number seized, varied, according to the difference in the period, and in the class of the individuals attacked. From the 10th to the 15th, it did not exceed one in eight; and was then chiefly confined to the bearers and other descriptions of Camp followers; from the 15th to the 19th, it increased to one in three and a half, and then occurred, principally amongst the Europeans and Sepoys; and from the 22d, to the 30th, it decreased greatly. We have said, that the disease was at its height on the 18th. Of the 2d Battalion 11th Native Infantry, 56 Sepoys were admitted on that day; from 20 to 30 on each succeeding day to the 22d, and from thence to the end of the month only 20 in all. Of the European Artillery, the average of deaths was one fourth; of Golundaz, rather more than a fifth; of gun lascars, less than a fifth; of drivers a third; and of Magazine men, a half. Of the Natives, the two former classes are chiefly Moossulmans, the two latter Hindoos. The great mortality amongst Europeans may be ascribed to their constitutions having been previously debilitated by irregular modes of living, and uncongenial climate; to the comparative severity of the attack, and greater struggle in a plethoric habit and muscular frame. Women were equally with men obnoxious to the disease; and died in a like proportion. Thus of 268 females attacked, 43, or one-sixth died. It was observed, that concubines and prostitutes suffered in a larger proportion than other descriptions of women: probably from their dissipated habits, and precarious mode of life.

The mortality in the Hansi Force was very inconsiderable. Not more than 271 persons in all were seized; of whom 51, or one in five and a half perished. Of these a large proportion was Sepoys; of whom 126 were attacked, and 27, or rather more than a fifth, died. The majority of deaths occurred in the early part of the disease; when it was more virulent than afterwards. Six Europeans only were attacked, of whom one died.

In the Rajpootana Force, which in fighting men and followers amounted perhaps to 15,000, the mortality was greater. The armed part of the Force consisted of 96 Europeans, (Officers not included) and 4,100 Natives; of whom 292 were admitted, and 122, nearly one half, died. The different Corps were variously affected. Of the European Artillery, only 3 were seized: all of whom recovered. Of the Goolundaz 240 in number, 6 sickened, and 2 died; of 250 Gun lascars. 21, of whom 12 died; of 392 Ordnance Drivers, 44, of whom 17 died; of 294 Pioneers, 53, of whom 23 died: Of the 2d Cavalry, 600 strong, 8, of whom 2 died; of the 2d Local Cavalry, 720 strong, 4, of whom 2 died; of 5 Companies 1st Battalion 27th Regiment, 18, of whom 7 died; of the 1st Battalion 28th Regiment, 944 strong, 135, of whom 57 died; and of 2d Battalion 19th Regiment, 887 strong, 3, of whom none died. Of 75 Camp followers who took medicine, 15 died. Here Europeans were slightly affected, and the lower classes suffered most. This was not uniformly the case; for according to one statement, the Sepoys suffered more than the camp followers in the 1st Battalion 28th Regiment. By this account, the strength of the Battalion is stated at 1,004; of whom fell ill 144, and died 60; whilst of 513 Camp followers, 35 only were seized, and 14 died: the casualties among the Sepoys being in double proportion to those of the followers. The Battalion consisted of 878 Hindoos, and 126 Mussulmen; of the former, three fifths were Bramins and Rajpoots; the remainder of low cast—57 Bramins were taken ill, and 30 died; Rajpoots, 47, and 16 died; low casts, 21, and 9 died: or of the whole affected, a little more than one half. Of the Mahometans, 19 were seized, and 5, or one fourth, died. So that the disease was more fatal to Bramins than to Rajpoots; to Rajpoots than to the lower casts; and to Mussulmans the least so of all. It was most destructive from the 14th to the 22d September; of 108 admitted from this Battalion in the 1st week of its visit, 51 died; after which the average was about one fourth.

In the Left Division, of 8,500 fighting men, 125 cases occurred, of whom 49, more than a third, died; 30 in April, 18 in May, and 1 in June. Here the disease equally attacked Hindoos and Mussulmans of all orders: Drivers, Lascars, Bearers, Grooms, and Grasscutters, not being more liable than the regular troops. Children were peculiarly exempt; females not. The mortality was greatest from the 10th to the 21st April.

In the Nagpore Force, from the 31st May to the 15th June, of about 4,000 regular troops, 13 Europeans were seized, of whom 6 died; 211 Sepoys, of whom 29, or one seventh, died. The mortality was greatest, perhaps two thirds, at first. The Camp followers were affected very severely; grooms more so than any others.—The Artillery lascars, drivers, and water-carriers were most exempt. Women were affected equally with men; and children at the breast were not safe from its attacks.

From the whole of the foregoing statements, we think it may perhaps be inferred: 1st. That the sum total of the mortality occasioned by the epidemic fell far short of the rate assigned to it by the voice of the public during the season of alarm. 2d. That the mortality was proportionately much greater, among large and dense, than among small and dispersed bodies of men. 3d. That in a given place, it was generally greater in the commencement, and middle, than towards the termination of the disorder. 4th. That when unlimited by the intervention of remedial means, it generally amounted to one half, and sometimes to two thirds of the seizures. 5th. That where medical aid was duly exhibited, it rarely amounted to one third, and was generally as low as one fifth of the attacked. 6th. That men were generally more susceptible than women; and that infants and children were nearly exempt." 184.

A section is dedicated to the description of some peculiarities of the disease. Of these peculiarities we shall select only the most peculiar, for our limits are already overstepped. It has been believed by many, that during its prevalence, cholera swallowed up all other diseases and reigned sole and despotic. This was not the case in Bengal, other complaints proceeding during its prevalence with little, if any, diminution or alteration. Relapses occasionally occurred during the progress of convalescence, but, after recovery was complete, instances of a second seizure were very uncommon indeed, so much so, as fully to establish the truth of the general rule to the contrary.

"Another curious circumstance in the economy of the disease, was, that, not only were persons, who had once undergone its attack, free from its further assaults; but even individuals, and bodies of men, who having come within its pestilential influence, had escaped unaffected, were nevertheless much less obnoxious to its future visits, than those who had not before been exposed to the virus. In other words a village, which was visited by the Epidemic during the first year of its prevalence, would, on the disease re-appearing in that part of the country, be much less likely to suffer, than another village, which had not before been affected; and an individual going from the former into the infected air of the latter, would have a better chance of immunity, than its inhabitants, who had not undergone the previous seasoning. This was the case, to a greater or less degree, in every part of the Provinces; in which it was generally remarked, that the Epidemic, on its recurrence, either did not at all revisit the places formerly affected, or only in a much lighter manner, than those, to which it was yet a stranger. In Tirhoot, particularly, in which the Epidemic twice appeared at two distant periods, the truth of this observation was strikingly illustrated; since, according to the information of a very intelligent observer, not a single instance occurred, of the disease revisiting the same place, throughout the whole extent of the district.

But it is in the different Divisions of the Army, the bodies composing which long remain under the eye of the same Medical Officers, that we should expect to find the existence of this law most clearly established. It is here, accordingly, that we have the best examples of its reality. Thus in the Jubbulpore Force, the 7th Regiment of Cavalry, and 2nd Battalion 13th Native Infantry, which Corps had suffered severely in November with the Centre Division, and at the Bridge of Boats, remained quite exempt. Thus, too, the 2nd Battalion 19th, which was violently affected by the disease in August, had only three slight cases in September; when the other corps of the Rajpootana force were so roughly visited. But the best illustrations are to be found in the Centre Division. When this force broke up after the termination of the campaign, His Majesty's 24th Regiment of Dragoons, and 87th Regiment of Foot, and the 1st Battalion 8th Regiment Native Infantry, marched to Cawnpore; where they were stationed in April and May, when the city and cantonment were suffering from the disease. At this time the 24th Dragoons remained quite free; His Majesty's 87th had two slight cases, among the recruits who had not been with the Centre Division, and no death; and the 1st Battalion 8th Regiment N. I. had, according to one statement, no case, according to another, one only, and according to a third, three or four, all slight attacks. The situation of the latter Corps was such, as to give additional proof of the immunity of bodies previously exposed not being accidental. For it so happened, that this Battalion was placed right between the 2nd Battalion 15th N. I. and Craigie's Levies; both of which suffered severely, as not having earned the same means of protection. Camp followers of all descriptions were equally exempt; and one person only, an European Officer, who had been with the Centre Division, fell a victim to the disorder. In like manner, the 2nd Battalion 25th Regiment Native Infantry, which again fell in with the disease in April, whilst marching from the Tiraee for Lucknow by Gorruckpore, then suffered comparatively little. It had indeed 25 cases and 5 deaths; but of these only one was a case of relapse or recurrence, and even in it the symptoms of both attacks were very mild. But a still more extraordinary instance occurred in Lord Hastings' camp, during the march to Gorruckpore, towards the latter part of the same month. The disease here first broke out among the followers of a gentleman, who had just joined the party; and in a few days attacked between 50 and 60, out of 400, chiefly of the class of bearers. It next got among the servants of several Gentlemen in the Civil Service then in attendance upon the Governor General; and to the period of its decline, was confined to such persons, as had not been with the Centre Division. This could not be explained on any difference of situation; for the party daily changed ground, and the new comers were mixed promiscuously with those, who had been previously exposed to infecion. Nay, it further appears, that after attacking the first party, the disease made a long stretch, and next shewed itself amongst other persons, not yet seasoned, in the opposite end of the line: leaving all between untouched. If any other proofs were necessary, we might cite the case of the 2nd Battalion 3d Regiment, the greater part of which, having had the disease at Shergurh, were not at all affected, although stationed at Banda, when the town suffered severely. But enough, we think, has been already said, to shew, that the human frame, on being exposed for some time to the pestilential virus, got habituated to it, and in a great measure became insusceptible of its malignant influence.

There is reason to believe, that the lower animals were in some measure affected by the corrupt state of the air at this period. For it was observed in many places, that an unusual mortality occurred amongst black cattle, sheep, dogs, and other domestic animals. Thus in the Backergunge District, cattle had the disorder, and were cured by opium and the other remedies found most serviceable in the human species. Cows when seized, shed their young. So, in Tipperah, great numbers of horned cattle and sheep were seized with vomiting and convulsions, and suddenly expired. In 1815 again, half the cattle of the lower part of Tipperah were carried off by a disease similar to Cholera. In Delhi, dogs died rapidly; and more horses than usual were carried off by the dry gripes.* In the Rajpootana Force, and throughout the whole of the Jeypore

* "In this city, a curious thing was, that large swarms of flies, which had infested the place before the breaking out of the Epidemic, wholly disappeared during its prevalence; and returned as it withdrew. This might be owing to the cold, sharp, westerly wind then blowing."

and Nagpore territories, the season was remarkably fatal to camels; and in the Centre Division, domestic animals of all descriptions died in great numbers; but in the latter instance the mortality might be ascribed to want of proper care and food. At Sumbhulpore, an Elephant had every symptom of Cholera; and was cured by brandy and laudanum. But the affection of brutes was by no means general. Thus in Dacca, Mymensing, Rajshahy, Nuddeea, Bhaugulpore, Tirhoot, Sarun, &c. the lower orders of animals are expressly stated to have enjoyed their usual health: So that the circumstance of their sickness in other quarters, during the prevalence of the Epidemic, may have been perfectly fortuitous." 195.

The last section is on the treatment of cholera. With this we need not occupy our readers. The following short summary conveys the result of the practice of our brethren in Bengal.

" 1. The disease sometimes attacked with such extreme violence, as from the commencement, apparently to place the sufferer beyond the reach of medical aid, and to render every curative means employed equally unavailing.

2. The difference in the degree of mortality amongst those, who did, and those who did not take medicine, was such, as to leave no doubt, that, when administered in time, and with discrimination, it frequently saved the patient from death.

3. The chances of a patient's receiving benefit from medicine, diminished in proportion with the increased duration of the attack.

4. In Europeans generally, and in robust Natives, bleeding could commonly be practised, where the patient was seen within one, two, or perhaps three hours, from the beginning of the attack; and in all cases, in which it was resorted to, under such favourable circumstances, it was more successful than any other remedy, in cutting short the disease: usually resolving spasm; allaying the irritability of the stomach and bowels; and removing the universal depression under which the system laboured.

5. Amongst the generality of Natives, the depressing influence of the disease was so powerful, and rapid in its operation, as almost immediately to produce complete collapse, and nearly destroy arterial action: and, therefore, to render venesection, for the most part, from the beginning, impracticable.

6. In such cases the cure was best attempted by diluents, powerful anodynes, and stimulants; combined with calomel; and followed up by mild laxatives, and tonics.

7. Although it cannot be affirmed, that calomel possessed any specific power in checking the disorder, it was undoubtedly frequently useful in soothing irritability; and was, perhaps, of more certain sedative operation than any other medicine." 247.

Here we conclude. We shall make few remarks. We have placed the substance of all the Indian Reports before our readers, and they may form, as we have formed, conclusions from them. The bulk of the profession being now put in possession of these data, may proceed to the investigation of the disease as it exists in our own country, or in others, less cramped by ignorance, less fettered by prejudice than they would have been without them. For our own parts, we declare that we had rather know nothing of the disease, as it first shewed itself in India, than know it through the medium of the imperfect, patched, and garbled statements, which have abused of late the public ear. We have dedicated some trouble, some time, and some space, to procuring and reviewing these documents, and we deem no apology necessary to our readers for their re-publication. On the contrary, we conceive that we have done a service to those who are anxious for sound information, correct facts, and narratives highly interesting to all, but particularly so to medical men.

